# Effectiveness of automated alerting system compared to usual care for the management of sepsis

**DOI:** 10.1038/s41746-022-00650-5

**Published:** 2022-07-19

**Authors:** Zhongheng Zhang, Lin Chen, Ping Xu, Qing Wang, Jianjun Zhang, Kun Chen, Casey M. Clements, Leo Anthony Celi, Vitaly Herasevich, Yucai Hong

**Affiliations:** 1 Department of Emergency Medicine, Key Laboratory of Precision Medicine in Diagnosis and Monitoring Research of Zhejiang Province, Sir Run Run Shaw Hospital, Zhejiang University School of Medicine, Hangzhou, China; 2 Department of Critical Care Medicine, Affiliated Jinhua Hospital, Zhejiang University School of Medicine, Jinhua, People’s Republic of China; 3 Emergency Department, Zigong Fourth People’s Hospital, Zigong, Sichuan China; 4 Institute of Medical Big Data, Zigong Academy of Artificial Intelligence and Big Data for Medical Science Artificial Intelligence, Zigong, Sichuan China; 5 Key Laboratory of Sichuan Province, Zigong, China; 6 Department of Surgery, University of Virginia, Charlottesville, VA USA; 7 Department of Emergency Medicine, Mayo Clinic, Rochester, MN USA; 8 Department of Biostatistics, Harvard T H Chan School of Public Health, Boston, USA; 9 Laboratory for Computational Physiology, Massachusetts Institute of Technology, Cambridge, USA; 10 Division of Pulmonary, Critical Care and Sleep Medicine, Beth Israel Deaconess Medical Center, Boston, USA; 11 Department of Anesthesiology and Perioperative Medicine, Division of Critical Care Medicine, Mayo Clinic, Rochester, MN USA

**Keywords:** Diagnosis, Infectious diseases

## Abstract

There is a large body of evidence showing that delayed initiation of sepsis bundle is associated with adverse clinical outcomes in patients with sepsis. However, it is controversial whether electronic automated alerts can help improve clinical outcomes of sepsis. Electronic databases are searched from inception to December 2021 for comparative effectiveness studies comparing automated alerts versus usual care for the management of sepsis. A total of 36 studies are eligible for analysis, including 6 randomized controlled trials and 30 non-randomized studies. There is significant heterogeneity in these studies concerning the study setting, design, and alerting methods. The Bayesian meta-analysis by using pooled effects of non-randomized studies as priors shows a beneficial effect of the alerting system (relative risk [RR]: 0.71; 95% credible interval: 0.62 to 0.81) in reducing mortality. The automated alerting system shows less beneficial effects in the intensive care unit (RR: 0.90; 95% CI: 0.73–1.11) than that in the emergency department (RR: 0.68; 95% CI: 0.51–0.90) and ward (RR: 0.71; 95% CI: 0.61–0.82). Furthermore, machine learning-based prediction methods can reduce mortality by a larger magnitude (RR: 0.56; 95% CI: 0.39–0.80) than rule-based methods (RR: 0.73; 95% CI: 0.63–0.85). The study shows a statistically significant beneficial effect of using the automated alerting system in the management of sepsis. Interestingly, machine learning monitoring systems coupled with better early interventions show promise, especially for patients outside of the intensive care unit.

## Introduction

Sepsis is a leading cause of mortality and morbidity in hospitalized patients^1,2^. A large body of published evidence shows the link between delayed responses including lactate measurement, antibiotic initiation, and fluid administration^3–5^ and adverse clinical outcomes^6^. Thus, the sepsis surviving campaign guideline recommends the prompt initiation of sepsis bundles for the treatment of sepsis in a variety of clinical settings^7^. With the rapid development of electronic health care records, the application of automated alerting systems to provide early warning for sepsis detection has triggered tremendous interest in the literature. There have been several prediction algorithms developed including rule-based and machine learning (ML) based methods. The former typically include those with standard SIRS or qSOFA criteria involving routine variables such as vital signs and laboratory findings. The latter utilized a variety of ML methods to alert sepsis including neural networks, random forests, and support vector machines. These methods are found to have high accuracy in predicting sepsis^8–11^. However, good statistical performance of a prediction model does not necessarily mean clinical usefulness of the model. It is more important for an automated alerting system to be able to improve patient-important outcomes. Thus, comparative effectiveness studies are mandatory to provide high-quality evidence for clinical decision-making.

There have been many studies exploring the clinical effectiveness of an automated alerting system for the management of sepsis^12,13^. Many investigators compared clinical outcomes between pre-and post-implementation of an automated system^14,15^. Systematic reviews evaluating the usefulness of automated alerting systems in sepsis have been reported in the literature. However, most of these studies evaluated reporting the diagnostic accuracy of the alerting system in predicting sepsis^12,16–18^, and a few evaluated the effectiveness in terms of clinically relevant outcomes, such as mortality and length of stay (LOS). For instance, Hwang and colleagues analyzed studies published between 2009 and 2018 and found that algorithm-based methods had high accuracy in predicting sepsis. To our knowledge, only one such analysis reported improved mortality outcome^19^. A systematic review conducted by the Cochrane collaboration included three RCTs and concluded that it was unclear what effect automated systems for monitoring sepsis have on clinical outcomes due to the low quality of included studies^13^. The number of comparative effectiveness studies has been steadily increasing in recent years with several new RCTs being reported^20,21^. Thus, an updated systematic review is needed to renew evidence for clinical practice. Furthermore, the results of these studies are conflicting due to differences in the prediction algorithm, clinical setting, and study designs. To address the heterogeneity of these studies and to appraise the evidence for clinical practice, we performed a systematic review to critically evaluate the quality of this evidence.

## Results

### Study selection

**Fig. 1:**
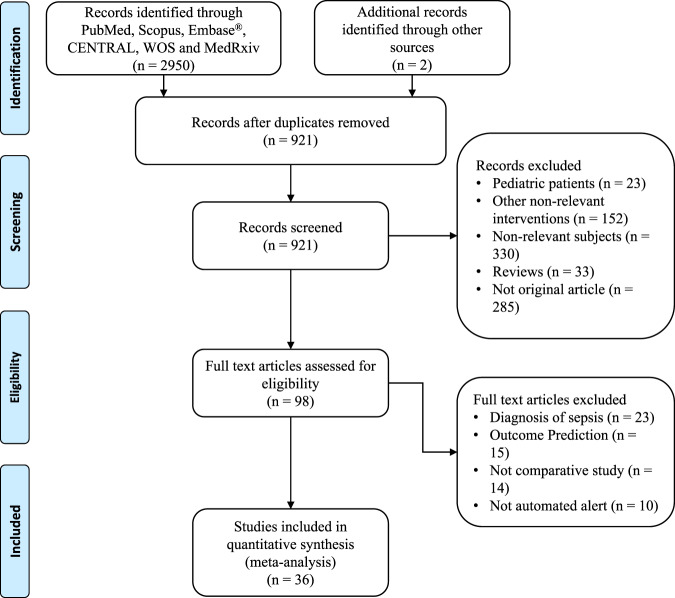
Flowchart of study selection. *WOS* web of science, *CENTRAL* Cochrane Central Register of Controlled Trials.

The initial search identified 2950 articles from the databases, and 921 were screened after the removal of duplicated items. A total of 823 citations were excluded by reviewing the title and abstract because they were pediatric patients, non-relevant interventions, reviews, and other non-original articles. The remaining 98 citations were further screened for the full text, and finally, we included 36 articles for quantitative analyses (Fig. 1). The number of publications were increasing until the year 2017 and then declined (Supplementary Fig. 1).

### Study characteristics

A total of 36 studies were included in the study, spanning from the year 2010 to 2021 (Table 1). There were 6 RCTs^20–25^ and 30 NRS^14,26–54^. Four studies explored ML-based prediction for sepsis/severe sepsis alert^22,26,27,30^. Six studies were conducted in ICU setting^14,22,23,25,44,51^. The sample sizes of RCTs ranged from 142 to 1123. Burdick’s study included 17,758 subjects because this study involved nine medical centers and all at-risk patients were analyzed for clinical outcomes^27^.

**Table 1:** Characteristics of included studies.

First author	Publication year	Country	Mean/median age (years)	Centers	Alert sending	Sample size	Population	Setting	Design	Method	Clinical intervention
Downey	2018	UK	65.2	Single	Nurse	226	Surgical wards	Wards	RCT	rule-based	sepsis alert
Downing	2019	USA	63	Single	Physician and nurse	1123	wards and stepdown unit	Wards	RCT	rule-based	severe sepsis alert
Hooper	2012	USA	55 vs. 54	Single	Physician	442	modified SIRS	ICU	RCT	rule-based	sepsis alert
Shimabukuro	2017	USA	59.3 vs. 58.9	Single	Physician	142	adult ICU patients	ICU	RCT	ML-based	severe sepsis alert
Semler	2015	USA	57 vs. 55	Single	ICU resident physician or nurse practitioner	407	ICU sepsis	ICU	RCT	rule-based	bundle implementation
Tarabichi	2021	USA		Single	Pharmacist	598	ED adult	ED	RCT	rule-based	sepsis alert
Arabi	2017	Saudi Arabia	NA	Single	Nurse	1330	sepsis or septic shock	ED	NRS	rule-based	bundle implementation
Austrian	2018	USA	67.1	Single	Nurse	2144	Hospitalized patients	Wards	NRS	rule-based	sepsis alert
Bader	2020	Jordan	NA	Single	Nurse	168	ED patients with cancer and sepsis	ED	NRS	rule-based	sepsis alert
Benson	2014	USA	59.8 vs. 60.1	Single	Nurse	239	SIRS patients	Wards	NRS	rule-based	sepsis alert
Berger	2010	USA	42.2 vs. 42.4	Single	NA	5796	ED adult	ED	NRS	rule-based	severe sepsis alert
Burdick	2020	USA	45	Multiple	Provider	17758	Sepsis-related patients	Wards	NRS	ML-based	severe sepsis alert
Manaktala	2017	USA	64 vs. 63	Single	Nurse	7388	Hospitalized patients	Wards	NRS	rule-based	sepsis alert
Mathews	2014	USA	NA	Single	NA	2109	Sepsis-related patients	Wards	NRS	rule-based	severe sepsis alert
McRee	2014	USA	70.5 vs. 69.9	Single	Nurse	171	Sepsis-related patients	Wards	NRS	rule-based	sepsis alert
McCoy	2017	USA	NA	Single	NA	1328	Hospitalized patients	Wards	NRS	ML-based	sepsis alert
Croft	2014	USA	NA	Single	Physician or extender	184	Surgical ICU	ICU	NRS	rule-based	bundle implementation
Gatewood	2015	USA	NA	Single	Provider	642	ED adult	ED	NRS	rule-based	sepsis alert
Ferreras	2015	Spain	77.6 vs. 75.5	Single	Nurse	433	Sepsis-related patients	ED	NRS	rule-based	sepsis alert
Giannini	2019	USA	58.5 vs. 58.7	Single	Covering care team	3677	non-ICU admissions	Wards	NRS	ML-based	severe sepsis alert
Guirgis	2017	USA	57.3 vs. 57.1	Single	Provider	3917	Hospitalized patients	Wards	NRS	rule-based	sepsis alert
Hayden	2016	USA	55 vs. 56	Single	Physician	238	Sepsis-related patients	ED	NRS	rule-based	bundle implementation
Honeyford	2020	UK	NA	Single	Doctors and nurses	21183	Sepsis-related patients	ED	NRS	rule-based	bundle implementation
Idrees	2016	Australia	58 vs. 59	Single	NA	90	sepsis or septic shock	ICU	NRS	rule-based	sepsis alert
Jung	2018	USA	54.7 vs. 50.6	Single	Physician and nurse	30	Sepsis	ICU	NRS	rule-based	Sepsis-alert
Lipatov	2022	USA	66.2 vs. 66.7	Single	NA	1950	MICU sepsis	ICU	NRS	rule-based	bundle implementation
Machado	2018	USA	68.5 vs. 67.7	Single	Physician, nurse, X-ray technician and pharmacist	314	ED patients with suspected sepsis	ED	NRS	rule-based	sepsis alert
Moore	2019	USA	NA	Single	Physician	312	ED patients with sepsis	ED	NRS	rule-based	bundle implementation
Na	2021	Korea	62 vs. 62	Single	Provider	1596	Hospitalized patients	Wards	NRS	rule-based	sepsis alert
Narayanan	2016	USA	66.7 vs. 67.1	Single	Provider	214	ED adult	ED	NRS	rule-based	severe sepsis alert
Sawyer	2011	USA	52.6 vs. 50.4	Single	Provider	270	Sepsis-related patients	Wards	NRS	rule-based	sepsis alert
Song	2019	Korea	74 vs. 77	Single	Nurse	631	ED patients with qSOFA>2	ED	NRS	rule-based	bundle implementation
Shah	2018	USA	61.5 vs. 67	Single	Physician	115	ED patients with sepsis	ED	NRS	rule-based	sepsis alert
Threatt	2020	USA	NA	Single	Physician	310	ED patients with sepsis	ED	NRS	rule-based	bundle implementation
Umscheid	2015	USA	62 vs. 59.7	Multiple	covering provider and rapid response coordinator	1140	non-ICU patients	Wards	NRS	rule-based	severe sepsis alert
Westra	2017	USA	64.1 vs. 62.7	Single	Nurse	778	Hospitalized patients	Wards	NRS	rule-based	sepsis alert

*ED* emergency department, *ICU* intensive care unit, *ML* machine learning, *SIRS* systematic inflammatory response syndrome, *qSOFA* quick sequential organ failure assessment, *NRS* non-randomized study.

### Risk of bias in studies

**Fig. 2:**
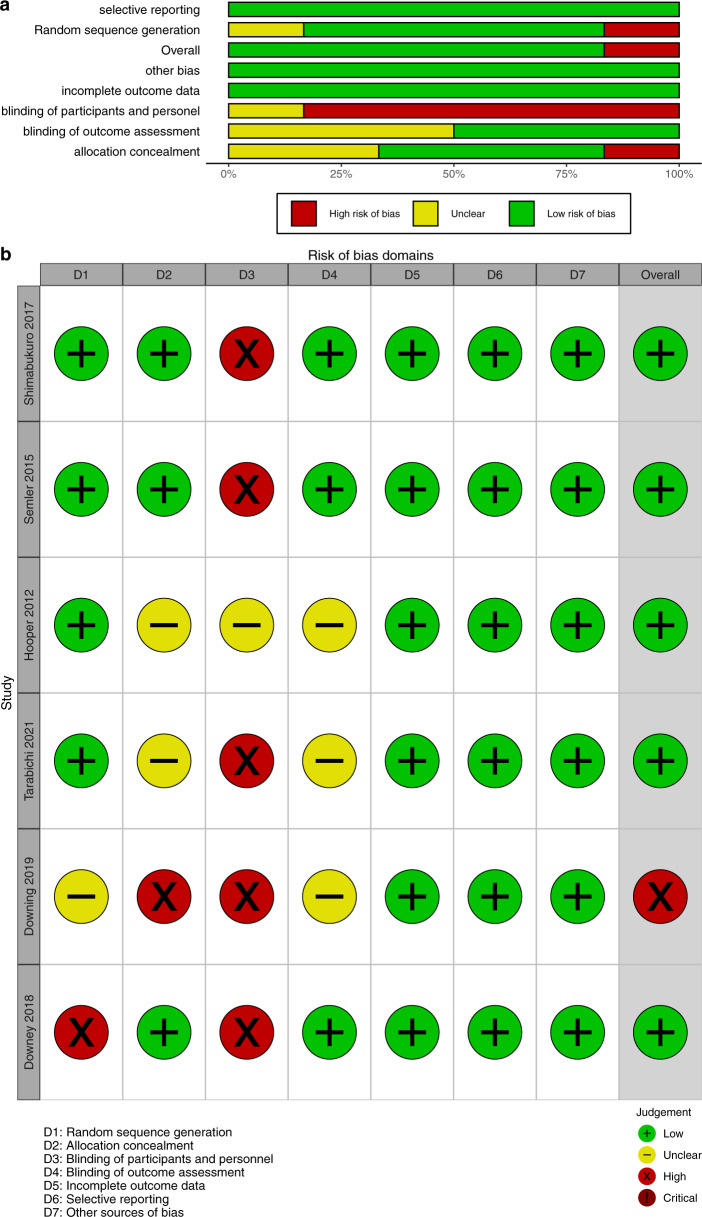
Risk of bias assessment for randomized controlled trials. **a** Summary statistics for the risk of bias assessment for RCT. **b** Risk of bias assessment for each RCT. *RCT* randomized controlled trial.

**Fig. 3:**
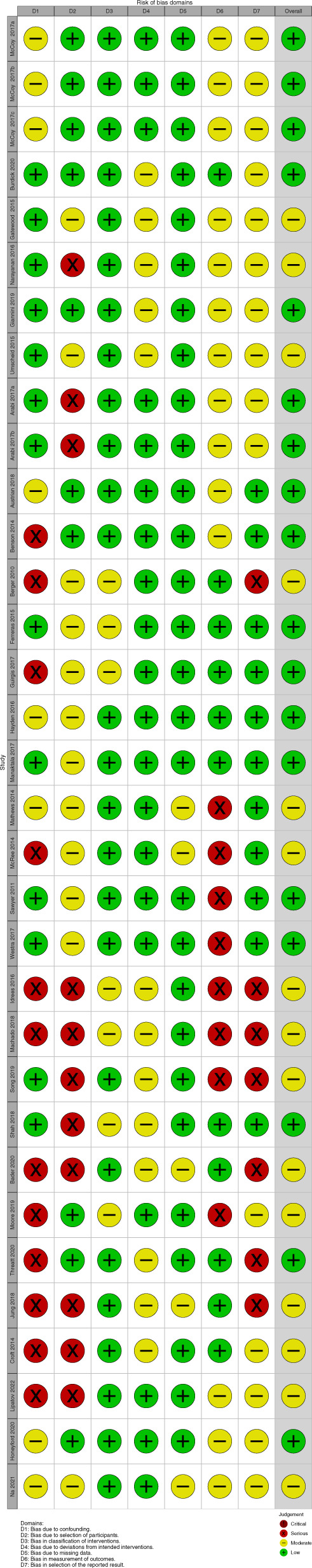
Risk of bias assessment for each of the non-randomized studies. The rows represent the individual study and the columns represent the quality items as annotated at the bottom.

**Fig. 4:**
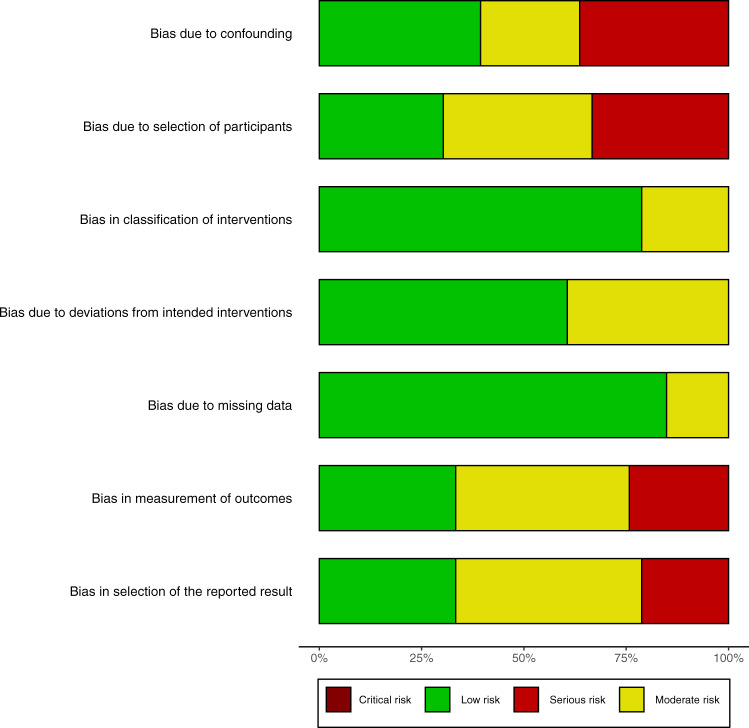
Summary of the risk of bias assessment for non-randomized studies. The bars show the percentage of studies with different levels of quality as indicated by colors.

The risk of bias was assessed with different tools for RCTs (Fig. 2) and NRS (Figs. 3 and 4). While some studies did not report all the necessary information to grade the methodology, the RCTs were found to have less risk of bias. NRS studies had more risk of bias in the selection of participants and outcome measurements.

### Results of syntheses

**Fig. 5:**
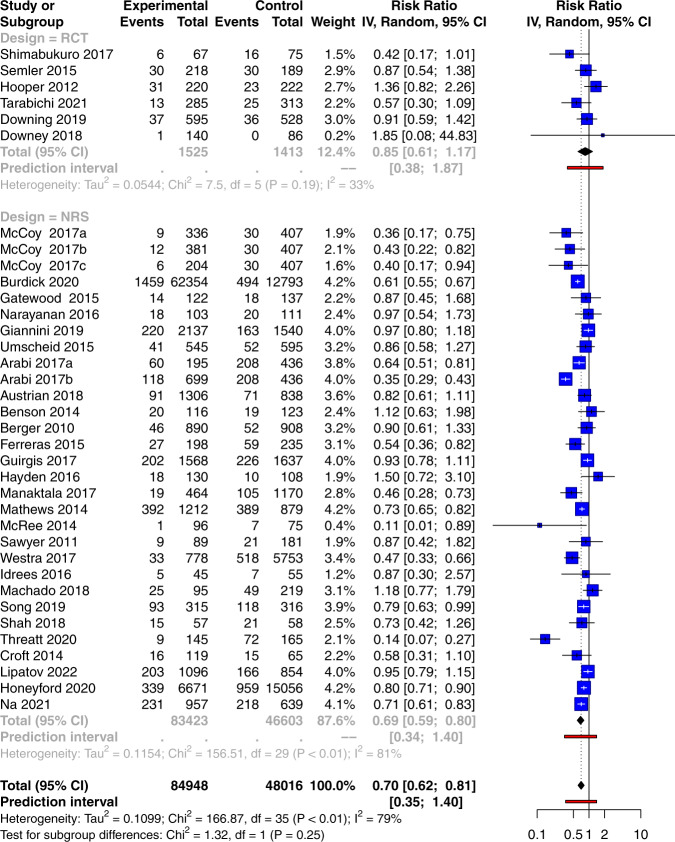
Forest plot for pooled effects of the automated alerting system in mortality outcome. The size of the blue square indicates the weight of each study. The black diamond represents the pooled effect size for each subgroup as well as for the overall effect. The red bars represent the prediction interval. *IV* inverse variance, *RCT* randomized controlled trial, *CI* confidence interval.

The mortality outcome reported in individual studies were inconsistent across studies (Fig. 5). While some studies reported beneficial effects^22,26,39^, others reported harmful effects of the automated alerting system^23,38,45^. By pooling risk ratios across RCTs, there is a trend toward improved mortality in the experimental group, but this does not reach statistical significance (RR: 0.85; 95% CI: 0.61–1.17). However, there was a statistically significant beneficial effect in the NRS (RR: 0.69; 95% CI: 0.59–0.80). While there was no statistically significant heterogeneity in RCTs (*I*^2^ = 33%, *p* = 0.19), there was significant heterogeneity across NRS (I^2^ = 81%, p < 0.01). In subgroup analysis, it was interesting to note that the automated alerting system had less beneficial effects in the ICU (RR: 0.90; 95% CI: 0.73–1.11) than that in ED (RR: 0.68; 95% CI: 0.51–0.90) and ward (RR: 0.71; 95% CI: 0.61–0.82; Supplementary Fig. 2). Furthermore, ML-based prediction methods showed a larger magnitude in reducing mortality (RR: 0.56; 95% CI: 0.39–0.80) than rule-based methods (RR: 0.73; 95% CI: 0.63–0.85; Supplementary Fig. 3). Bundle recommendation alerting (RR: 0.63; 95% CI: 0.43–0.94; Supplementary Fig. 4) performed better than sepsis alert in reducing mortality (RR: 0.78; 95% CI: 0.66–0.92; Supplementary Fig. 4). Bayesian meta-analysis of RCTs with NRS as prior showed that automated alert was able to reduce the mortality risk (RR: 0.71; 95% credible interval: 0.62 to 0.81; Supplementary Fig. 5).

ICU length of stay was reported in 11 studies and there was no evidence that automated alerts could significantly reduce the length of stay in ICU (MD: -1.33; 95% CI: -3.34 to 0.67). There was also substantial heterogeneity across these studies (*I*^2^ = 97%, *p* < 0.01; Supplementary Fig. 6). Other subgroup analyses failed to find factors to explain the heterogeneity (Supplementary Figs. 7–9). Hospital length of stay was reported in 21 studies. Overall, there was a significant reduction in hospital length of stay (MD: -2.42; 95% CI: -4.43 to -0.41), with substantial heterogeneity across studies (*I*^2^ = 94%, *p* < 0.01). The heterogeneity could not be fully explained by the study design (Supplementary Fig. 10). However, studies conducted in hospital wards showed more consistent results (*I*^2^ = 77%, *p* < 0.01; Supplementary Fig. 11). The methods (ML or rule-based), specific rules (SIRS, qSOFA, and MEWS) used to alert sepsis, and purposes of alerting were not able to account for the heterogeneity (Supplementary Figs. 12 to 14).

### Reporting biases

**Fig. 6:**
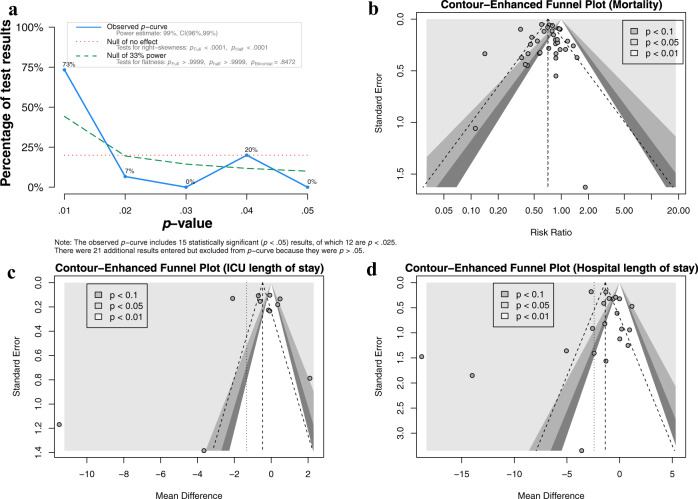
Assessment of publication bias. **a** Visual inspection of the p-curve plot shows a right-skewed distribution with 73% of the *p*-values between 0 and 0.01 and only 20% of *p*-values between 0.03 and 0.05. The statistical tests against the null hypothesis that all of the significant p-values are false positives are highly significant. Thus, at least some of the p-values are likely to be true positives. Finally, the power estimate is very high, 99%, with a tight confidence interval ranging from 96% to 99%. Somewhat redundant with this information, the p-curve also provides a significant test for the hypothesis that power is less than 33%. This test is not significant, which is not surprising given the estimated power of 99%. The contour-enhanced funnel plots showed significant levels area at 0.1, 0.05, and 0.01 for **b** mortality, **c** ICU length of stay, and **d** hospital length of stay. Some studies appeared to be missing in areas of high statistical significance, thus it is possible that the asymmetry is not due to publication bias. *ICU* intensive care unit.

The reporting biases of included studies were assessed by p-curve, which showed a right-skewed distribution with 73% of the p-values between 0 and .01 (Fig. 6). The statistical tests against the null hypothesis that all the significant p-values are false positives were rejected with high statistical significance. Thus, at least some of the p-values are likely to be true positives. Finally, the power estimate is very high, 99%, with a confidence interval ranging from 96% to 99%. The contour-enhanced funnel plots showed that the distributions of studies were generally symmetric for mortality and hospital LOS (Fig. 6). The supposed missing studies are in the area of high statistical significance; thus, it is possible that the asymmetry is not due to publication bias.

## Discussion

This study provides systematically updated evidence on the effectiveness of automated alerts for the management of sepsis in various settings. The results show that the management of sepsis with an automated alerting system can reduce the mortality rate, which is further confirmed by the Bayesian meta-analytic approach. Although there is no evidence that the automated alerting system can reduce ICU LOS, the hospital LOS is significantly reduced. Subgroup analyses indicate that the beneficial effect of automated alerting systems is less significant in ICU settings than that in ED and general wards. ML-based alerting systems appear to provide additional benefits as compared to rule-based methods.

The main finding in our study is that an automated alerting system can reduce mortality risk, probably attributable to the increased awareness of the sepsis onset. There has been a large body of evidence showing that early recognition of sepsis and prompt initial of sepsis bundle are associated with improved outcomes. For example, the reduction in time-to-antibiotic use is consistently reported to be associated with improved survival outcomes^55,56^. The same effects are also observed in other bundle components such as lactate measurement and fluid administration^57^. The effect of the automated alerting system is more prominent in the general ward and emergency setting than that in the ICU setting. Probably, ICU is already equipped with advanced monitoring modality, and physicians and nurses are in high acuity for sepsis surveillance as their usual care. The addition of a further automated alerting system will not provide further benefits.

The findings of the study have several novelties and clinical implications. First, the Bayesian meta-analytic approach was employed to integrate evidence from both RCTs and NRS. Although RCT is the gold standard design for comparative effectiveness, these data are sparse, smaller, and potentially unrepresentative of the patient populations or conditions found in real-world settings. Thus, real-world evidence from routine clinical practice provided by NRS is important to complement information from RCTs and potentially cover the ‘efficacy-effectiveness’ gap^58^. The results from the Bayesian meta-analysis are consistent with that from the frequentist meta-analytic approach.

Second, more in-depth subgroup analyses were performed to explore potential heterogeneity in component studies. Our analysis found that automated alerting systems deployed in ICU settings had less beneficial effects as compared to other settings. This is not surprising since ICU patients are already monitored closely by both automated systems and additional, attentive staff. In contrast, general wards and ED are equipped with much less staff, and some deteriorating conditions may not be recognized as quickly. In such situations, the use of an automated alerting system can have additional benefits to improve clinical outcomes. In line with this finding, other early warning scores have been widely deployed outside ICUs to improve the early recognition of deteriorating conditions^59^. Real-time automated alerting systems based on EMRs could help identify unstable patients, and early detection and intervention with the system may improve patient outcomes^60^.

Third, ML-based methods appear to be superior to other rule-based methods in improving mortality. ML-based methods estimate the presence of sepsis/severe sepsis by utilizing a higher number of relevant data points/biomarkers and can better capture the non-linear relationships between these variables^61,62^. Such complex relationships cannot be recognized to the same degree by humans. Rule-based methods are mostly based on established diagnostic criteria for identifying sepsis and sepsis is usually already present when a warning is triggered. Thus, the timeliness of diagnosis might be more easily achieved by using ML methods^15^.

Several limitations of the current study must be acknowledged. First, the qualities of included RCTs were variable. The blinding is difficult to achieve due to the nature of the intervention. The changes in medical decision-making dictated by the alerting are not necessarily well characterized. Additionally, the reported beneficial effects in the intervention group could be biased because clinicians know the allocation, and more attention may have been given to patients in the intervention group. Second, most component studies are NRS, which are prone to both measured and unmeasured confounding factors. The biased effect size from NRS was partly overcome by using a Bayesian meta-analytic approach. Third, there is remarkable heterogeneity among these included studies, which cannot be explained by some prespecified variables. Thus, more homogeneous large RCTs are needed to provide high-quality evidence^63^. Finally, machine-learning algorithms are sensitive to changes in the environment and subject to performance decay^64^. Continuous monitoring and updating are required to ensure their long-term safety and effectiveness. Healthcare processes of sepsis can change with accumulating evidence, requiring the ML algorithms to adapt to the new environment.

In conclusion, the study shows a beneficial effect of an automated alerting system in the management of sepsis. Interestingly, machine learning monitoring systems coupled with better early interventions show promise, especially for patients outside of ICUs. However, there is substantial heterogeneity and risk of bias across component studies. Further experimental trials are still required to improve the quality of evidence.

## Methods

### Eligibility criteria

Studies comparing the effectiveness of automated alerting systems for the management of sepsis were potentially eligible. The study population included hospitalized patients who were at risk for sepsis or patients who had sepsis. Patients who were at risk for sepsis were defined as per the original studies, including those presented to the emergency department (ED), general hospitalized patients, and ICU patients. Patients who were not initially in ICU and subsequently transferred to ICU due to deteriorated conditions were also included. The intervention was an automated alerting system integrated into the electronic healthcare records. The algorithms for the alerting system included ML-based methods and rule-based methods. The control group received usual care in which the medical providers would not receive any alerting messages. The outcomes included hospital mortality, LOS in the intensive care unit (ICU), and hospital. Evidence from non-randomized studies (NRS) was pooled with those from RCTs using the Bayesian meta-analytic approach. Subgroup analyses stratified by study design, setting, and methods of the alerting system were performed. The study protocol was registered in the International Prospective Register of Systematic Reviews (PROSPERO: CRD42022299219).

### Information sources

Electronic databases of PubMed, Scopus, Embase^®^, Cochrane Central Register of Controlled Trials (CENTRAL) and ISI Web of science, and MedRvix were searched from inception (the earliest year could date back to 1917) to December 2021. The reference lists of identified articles were also searched manually to identify additional references.

### Search strategy

Key terms related to (1) sepsis (sepsis or septic shock or septicemia), (2) automated alert (automated, ML, prediction, warning, and recognition), (3) clinical outcomes (mortality, length of stay), and (4) study design (randomized, controlled, pre-implementation and post-implementation) were searched in the databases. The type of literature was restricted to articles if a search engine had filtering functionality (Supplementary Methods).

### Selection process

Two authors (L.C. and P.X.) independently performed the literature selection process. The duplicated references from each database were removed by using the *RefManageR* package (version: 1.3.0). The title and abstract of each reference were firstly screened to remove some irrelevant articles such as reviews, animal studies, non-relevant interventions (such as antimicrobial susceptibility testing), irrelevant subjects (such as delirium management and prediction of AKI), pediatric patients (age < 16 years old), and case reports. The full-text articles were then screened for the remaining references. Conflicting results were solved in a meeting participated by all the review authors.

### Data collection process

A custom-made data collection form was prepared for data collection. Data includes the name of the first author, publication year, sample size, study design, prediction algorithm, number of patients in the intervention and control aims, the summary effect of the length of stay, and relevant standard deviation or interquartile range. Studies were classified into RCT and NRS by the design. NRS included those comparing patients managed with the automated alerting system versus historical controls. The studies might report mortality at different follow-up time points. If a study reported several mortality time points, we extracted the mortality in the hospital. The prediction algorithm was classified into rule-based or ML-based methods. The rule-based method referred to those using existing sepsis diagnostic criteria for the warning of the presence of sepsis. Two authors independently extracted data. Any conflicting results were solved by a third reviewer (Z.Z.).

### Assessment of risk of bias

The risk of bias was assessed separately for RCT and NRS. RCT was assessed from six aspects including sequence generation, allocation concealment, blinding of participants and personnel, blinding of outcome assessment, incomplete outcome data, selective reporting, and other sources of bias^65^. The risk of bias of NRS was assessed using the Risk Of Bias In Non-randomized Studies - of Interventions (ROBINS-I) tools, which included several aspects of bias due to confounding, bias due to selection of participants, bias in classification of interventions, Bias due to deviations from intended interventions, Bias due to missing data, Bias in the measurement of outcomes, Bias in the selection of the reported result^66^. The risk of bias assessment was performed independently by two reviewers (L.C. and K.C.) and any conflicting results were settled by a third opinion (Z.Z.).

### Effect measures and synthesis methods

The primary outcome was mortality and we reported risk ratio (RR) and confidence interval as the effect measure. The LOS in the hospital and ICU were reported as mean difference (MD). The evidence from NRS and RCT were pooled separately by using a conventional frequentist meta-analytic approach with the R *meta* package (version: 5.1-1)^67^. Due to the heterogeneity of the component studies, the random-effects method was employed to pool the effect measures. The Mantel-Haenszel estimator was used in the calculation of the between-study heterogeneity statistic Q which was used in the DerSimonian-Laird estimator^68^. Evidence from NRS was pooled with those from RCTs using the Bayesian meta-analytic approach. The effect measures of the NRS were used as the prior distribution for Bayesian meta-analysis for integrating RCT data^69^. This approach will ‘pull’ the treatment-effect estimates from the RCTs toward the summary effects from the NRS. Subgroup analyses stratified by setting (ICU, ED, or ward), methods of the alerting system (ML-based versus rule-based), and alerting purpose (bundle compliance, sepsis/severe sepsis alert) were performed.

### Reporting bias assessment

The reporting bias of included component studies was assessed and visualized using contour-enhanced funnel plots, which included colors to signify the significance level of each study in the plot. The significance level helps to differentiate asymmetry due to publication bias from that due to other factors^70^. *P*-curve analysis was also performed to detect *p*-hacking and publication bias in meta-analyses^71^. If the set of studies contains mostly studies with true effects that have been tested with moderate to high power, there are more *p*-values between 0 and 0.01 than between 0.04 and 0.05. This pattern has been called a right-skewed distribution by the *p*-curve authors. If the distribution is flat or left-skewed (more p-values between 0.04 and 0.05 than between 0 and 0.01), the results are more consistent with the null hypothesis than with the presence of a real effect.

### Reporting summary

Further information on research design is available in the Nature Research Reporting Summary linked to this article.

## Supplementary information

Supplementary Information

Reporting Summary

## Data Availability

All data were available in the original publications, and are available from the corresponding author on reasonable request.

## Code availability

Code for the analysis is available upon reasonable request from the corresponding author.

## References

[1] Herrán-Monge R (2019). Epidemiology and changes in mortality of sepsis after the implementation of surviving sepsis campaign guidelines. J. Intensive Care Med..

[2] Yu Y (2021). Effectiveness of anisodamine for the treatment of critically ill patients with septic shock: a multicentre randomized controlled trial. Crit. Care.

[3] Han X (2021). Identifying high-risk subphenotypes and associated harms from delayed antibiotic orders and delivery. Crit. Care Med..

[4] Seymour CW (2017). Delays from first medical contact to antibiotic administration for sepsis. Crit. Care Med..

[5] Ma P (2021). Individualized resuscitation strategy for septic shock formalized by finite mixture modeling and dynamic treatment regimen. Crit. Care.

[6] Han X (2018). Implications of centers for medicare & medicaid services severe sepsis and septic shock early management bundle and initial lactate measurement on the management of sepsis. Chest.

[7] Evans, L. et al. Surviving sepsis campaign: international guidelines for management of sepsis and septic shock 2021. *Crit. Care Med.*10.1097/CCM.0000000000005337 (2021). 10.1097/CCM.0000000000005337 34605781

[8] Nemati S (2018). An interpretable machine learning model for accurate prediction of sepsis in the ICU. Crit. Care Med..

[9] Diktas H (2020). A novel id-iri score: development and internal validation of the multivariable community acquired sepsis clinical risk prediction model. Eur. J. Clin. Microbiol Infect. Dis..

[10] Shakeri, E., Mohammed, E. A., Shakeri H. A., Z. & Far, B. Exploring features contributing to the early prediction of sepsis using machine learning. *Annu. Int. Conf. IEEE Eng. Med. Biol. Soc.* **2021**, 2472–2475 (2021). 10.1109/EMBC46164.2021.9630317 34891780

[11] Zhou, A., Raheem, B. & Kamaleswaran, R. OnAI-Comp: an online ai experts competing framework for early sepsis detection. *IEEE/ACM Trans. Comput. Biol. Bioinform.* **PP**, (2021). 10.1109/TCBB.2021.3122405 PMC10975783 34699366

[12] Makam AN, Nguyen OK, Auerbach AD (2015). Diagnostic accuracy and effectiveness of automated electronic sepsis alert systems: a systematic review. J. Hosp. Med..

[13] Warttig S (2018). Automated monitoring compared to standard care for the early detection of sepsis in critically ill patients. Cochrane Database Syst. Rev..

[14] Jung AD (2018). Sooner is better: use of a real-time automated bedside dashboard improves sepsis care. J. Surg. Res..

[15] Tran NK (2020). Novel application of an automated-machine learning development tool for predicting burn sepsis: proof of concept. Sci. Rep..

[16] Wulff A, Montag S, Marschollek M, Jack T (2019). Clinical decision-support systems for detection of systemic inflammatory response syndrome, sepsis, and septic shock in critically Ill patients: a systematic review. Methods Inf. Med..

[17] Alberto L, Marshall AP, Walker R, Aitken LM (2017). Screening for sepsis in general hospitalized patients: a systematic review. J. Hosp. Infect..

[18] Joshi M (2019). Digital alerting and outcomes in patients with sepsis: systematic review and meta-analysis. J. Med. Internet Res..

[19] Hwang MI, Bond WF, Powell ES (2020). Sepsis alerts in emergency departments: a systematic review of accuracy and quality measure impact. West J. Emerg. Med..

[20] Tarabichi, Y. et al. Improving timeliness of antibiotic administration using a provider and pharmacist facing sepsis early warning system in the emergency department setting: a randomized controlled quality improvement initiative. *Critical Care Med.*10.1097/CCM.0000000000005267 (2021). 10.1097/CCM.0000000000005267 34415866

[21] Downing NL (2019). Electronic health record-based clinical decision support alert for severe sepsis: a randomised evaluation. BMJ Qual. Saf..

[22] Shimabukuro DW, Barton CW, Feldman MD, Mataraso SJ, Das R (2017). Effect of a machine learning-based severe sepsis prediction algorithm on patient survival and hospital length of stay: a randomised clinical trial. BMJ Open Respir. Res.

[23] Hooper MH (2012). Randomized trial of automated, electronic monitoring to facilitate early detection of sepsis in the intensive care unit*. Crit. Care Med..

[24] Downey C, Randell R, Brown J, Jayne DG (2018). Continuous versus intermittent vital signs monitoring using a wearable, wireless patch in patients admitted to surgical wards: pilot cluster randomized controlled trial. J. Med. Internet Res..

[25] Semler MW (2015). An electronic tool for the evaluation and treatment of sepsis in the ICU: a randomized controlled trial. Crit. Care Med.

[26] McCoy A, Das R (2017). Reducing patient mortality, length of stay and readmissions through machine learning-based sepsis prediction in the emergency department, intensive care unit and hospital floor units. BMJ Open Qual..

[27] Burdick, H. et al. Effect of a sepsis prediction algorithm on patient mortality, length of stay and readmission: a prospective multicentre clinical outcomes evaluation of real-world patient data from US hospitals. *BMJ Health Care Inf.* **27**, (2020). 10.1136/bmjhci-2019-100109 PMC7245419 32354696

[28] Gatewood MO, Wemple M, Greco S, Kritek PA, Durvasula R (2015). A quality improvement project to improve early sepsis care in the emergency department. BMJ Qual. Saf..

[29] Narayanan N, Gross AK, Pintens M, Fee C, MacDougall C (2016). Effect of an electronic medical record alert for severe sepsis among ED patients. Am. J. Emerg. Med..

[30] Giannini HM (2019). A machine learning algorithm to predict severe sepsis and septic shock: development, implementation, and impact on clinical practice. Crit. Care Med..

[31] Umscheid CA (2015). Development, implementation, and impact of an automated early warning and response system for sepsis. J. Hosp. Med..

[32] Arabi YM (2017). The impact of a multifaceted intervention including sepsis electronic alert system and sepsis response team on the outcomes of patients with sepsis and septic shock. Ann. intensive care.

[33] Austrian JS, Jamin CT, Doty GR, Blecker S (2018). Impact of an emergency department electronic sepsis surveillance system on patient mortality and length of stay. J. Am. Med. Inform. Assoc.: JAMIA.

[34] Benson L, Hasenau S, O’Connor N, Burgermeister D (2014). The impact of a nurse practitioner rapid response team on systemic inflammatory response syndrome outcomes. Dimens Crit. Care Nurs..

[35] Berger T, Birnbaum A, Bijur P, Kuperman G, Gennis P (2010). A computerized alert screening for severe sepsis in emergency department patients increases lactate testing but does not improve inpatient mortality. Appl Clin. Inf..

[36] Ferreras JM (2015). Implementation of an automatic alarms system for early detection of patients with severe sepsis. Enferm. Infecc. Microbiol Clin..

[37] Guirgis FW (2017). Managing sepsis: electronic recognition, rapid response teams, and standardized care save lives. J. Crit. care.

[38] Hayden GE (2016). Triage sepsis alert and sepsis protocol lower times to fluids and antibiotics in the ED. Am. J. Emerg. Med.

[39] Manaktala S, Claypool SR (2017). Evaluating the impact of a computerized surveillance algorithm and decision support system on sepsis mortality. J. Am. Med. Inform. Assoc.: JAMIA.

[40] Mathews, K., Budde, J., Glasser, A., Lorin, S. & Powell, C. 972: Impact of an in-patient electronic clinical decision support tool on sepsis-related mortality. *Critic. Care Med.* **42**, (2014).

[41] McRee L, Thanavaro JL, Moore K, Goldsmith M, Pasvogel A (2014). The impact of an electronic medical record surveillance program on outcomes for patients with sepsis. Heart Lung.

[42] Sawyer AM (2011). Implementation of a real-time computerized sepsis alert in nonintensive care unit patients. Crit. Care Med..

[43] Westra BL, Landman S, Yadav P, Steinbach M (2017). Secondary analysis of an electronic surveillance system combined with multi-focal interventions for early detection of sepsis. Appl. Clin. Inform..

[44] Idrees M, Macdonald SP, Kodali K (2016). Sepsis Early Alert Tool: Early recognition and timely management in the emergency department. Emerg. Med. Australas.: EMA.

[45] Machado SM, Wilson EH, Elliott JO, Jordan K (2018). Impact of a telemedicine eICU cart on sepsis management in a community hospital emergency department. J. Telemed. telecare.

[46] Song, J. et al. The effect of the intelligent sepsis management system on outcomes among patients with sepsis and septic shock diagnosed according to the sepsis-3 definition in the emergency department. *J. Clin. Med.* **8**, (2019). 10.3390/jcm8111800 PMC6912745 31717855

[47] Shah T, Sterk E, Rech MA (2018). Emergency department sepsis screening tool decreases time to antibiotics in patients with sepsis. Am. J. Emerg. Med..

[48] Bader MZ, Obaid AT, Al-Khateb HM, Eldos YT, Elaya MM (2020). Developing adult sepsis protocol to reduce the time to initial antibiotic dose and improve outcomes among patients with cancer in emergency department. Asia-Pac. J. Oncol. Nurs..

[49] Moore WR, Vermuelen A, Taylor R, Kihara D, Wahome E (2019). Improving 3-hour sepsis bundled care outcomes: implementation of a nurse-driven sepsis protocol in the emergency department. J. Emerg. Nurs..

[50] Threatt DL (2019). Improving sepsis bundle implementation times: a nursing process improvement approach. J. Nurs. care Qual..

[51] Croft CA (2014). Computer versus paper system for recognition and management of sepsis in surgical intensive care. J. Trauma Acute Care Surg..

[52] Lipatov K (2022). Implementation and evaluation of sepsis surveillance and decision support in medical ICU and emergency department. Am. J. Emerg. Med..

[53] Honeyford K (2020). Evaluating a digital sepsis alert in a London multisite hospital network: a natural experiment using electronic health record data. J. Am. Med Inf. Assoc..

[54] Na SJ, Ko R-E, Ko MG, Jeon K (2021). Automated alert and activation of medical emergency team using early warning score. J. Intensive Care.

[55] Im Y (2022). Time-to-antibiotics and clinical outcomes in patients with sepsis and septic shock: a prospective nationwide multicenter cohort study. Crit. Care.

[56] Sterling SA, Miller WR, Pryor J, Puskarich MA, Jones AE (2015). The impact of timing of antibiotics on outcomes in severe sepsis and septic shock: a systematic review and meta-analysis. Crit. Care Med.

[57] Pepper DJ (2019). Antibiotic- and fluid-focused bundles potentially improve sepsis management, but high-quality evidence is lacking for the specificity required in the centers for medicare and medicaid service’s sepsis bundle (SEP-1). Crit. Care Med.

[58] Eichler H-G (2011). Bridging the efficacy-effectiveness gap: a regulator’s perspective on addressing variability of drug response. Nat. Rev. Drug Disco..

[59] McGaughey J, Fergusson DA, Van Bogaert P, Rose L (2021). Early warning systems and rapid response systems for the prevention of patient deterioration on acute adult hospital wards. Cochrane Database Syst. Rev..

[60] You S-H (2021). Incorporating a real-time automatic alerting system based on electronic medical records could improve rapid response systems: a retrospective cohort study. Scand. J. Trauma Resusc. Emerg. Med..

[61] Zhang Z (2016). A gentle introduction to artificial neural networks. Ann. Transl. Med..

[62] Greener, J. G., Kandathil, S. M., Moffat, L. & Jones, D. T. A guide to machine learning for biologists. *Nat. Rev. Mol. Cell Biol.*10.1038/s41580-021-00407-0 (2021). 10.1038/s41580-021-00407-0 34518686

[63] Arabi YM (2021). Electronic early notification of sepsis in hospitalized ward patients: a study protocol for a stepped-wedge cluster randomized controlled trial. Trials.

[64] Feng J (2022). Clinical artificial intelligence quality improvement: towards continual monitoring and updating of AI algorithms in healthcare. npj Digital Med..

[65] Higgins JPT (2011). The Cochrane Collaboration’s tool for assessing risk of bias in randomised trials. BMJ.

[66] Sterne JA (2016). ROBINS-I: a tool for assessing risk of bias in non-randomised studies of interventions. BMJ.

[67] Balduzzi S, Rücker G, Schwarzer G (2019). How to perform a meta-analysis with R: a practical tutorial. Evid. Based Ment. Health.

[68] Greenland S, Robins JM (1985). Estimation of a common effect parameter from sparse follow-up data. Biometrics.

[69] Sarri, G. et al. Framework for the synthesis of non-randomised studies and randomised controlled trials: a guidance on conducting a systematic review and meta-analysis for healthcare decision making. *BMJ EBM* bmjebm-2020-111493 10.1136/bmjebm-2020-111493 (2020). 10.1136/bmjebm-2020-111493 PMC8961747 33298465

[70] Peters JL, Sutton AJ, Jones DR, Abrams KR, Rushton L (2008). Contour-enhanced meta-analysis funnel plots help distinguish publication bias from other causes of asymmetry. J. Clin. Epidemiol..

[71] Simonsohn U, Nelson LD, Simmons JP (2014). p-Curve and effect size: correcting for publication bias using only significant results. Perspect. Psychol. Sci..

