# Supplementary material for: Effectiveness of automated alerting system compared to usual care for the management of sepsis
Source: NPJ Digit Med. 2022 Jul 19;5:101. doi: 10.1038/s41746-022-00650-5 (PMC9296632; doi:10.1038/s41746-022-00650-5)
Supplement: Supplementary file 1 — Supplementary Information [file 41746_2022_650_MOESM1_ESM.pdf]

Supplemental digital content for
Effectiveness of automated alerting system compared to usual care for the management of sepsis: a systematic review and meta-analysis

Table of Contents

Supplementary Methods **... 2**

Searching strategy in relevant databases 2

Supplementary Figure 1 Trends of the number of publications over time stratified by study design. 4

Supplementary Figure 2. Subgroup analysis for mortality outcome stratified by setting. 5

Supplementary Figure 3. Subgroup analysis for mortality outcome stratified by alerting method. 6

Supplementary Figure 4. Subgroup analysis for mortality outcome stratified by purpose of alerting. 7

Supplementary Figure 5. Bayesian meta-analysis of RCTs for mortality. 8

Supplementary Figure 6. Subgroup analysis for ICU length of stay stratified by the study design. 9

Supplementary Figure 7. Subgroup analysis for ICU length of stay stratified by the study setting. 10

Supplementary Figure 8. Subgroup analysis for ICU length of stay stratified by prediction methods. 11

Supplementary Figure 9. Subgroup analysis for ICU length of stay stratified by prediction purpose. 12

Supplementary Figure 10. Subgroup analysis for hospital length of stay stratified by study design. 13

Supplementary Figure 11. Subgroup analysis for hospital length of stay stratified by study setting. 14

Supplementary Figure 12. Subgroup analysis for hospital length of stay stratified by alerting methods. 16

Supplementary Figure 13. Subgroup analysis for hospital length of stay stratified by alerting purpose. 17

Supplementary Figure 14. Subgroup analysis for mortality stratified by different rules to alert sepsis onset. 18

Supplementary Methods

Searching strategy in relevant databases

The following searching strategy was applied on December 2021

Searching strategy in PubMed

Item number	Searching words	Number of items
1#	((sepsis[Title/Abstract]) AND (septic[Title/Abstract])) AND (septicemia[Title/Abstract])	161461
2#	early warning[Title/Abstract] OR machine learning[Title/Abstract] OR prediction[Title/Abstract] OR artificial intelligence[Title/Abstract] OR detection [Title/Abstract]	1310072
3#	mortality[Title/Abstract] OR length of stay[Title/Abstract]	910594
4#	randomized[Title/Abstract] OR controlled[Title/Abstract] OR observational[Title/Abstract] OR pre-implementation[Title/Abstract] OR pre-post [Title/Abstract]	1374458
5#	1# AND 2# AND 3# AND 4#	394

SCOPUS (n = 498)

TITLE-ABS-KEY (sepsis OR septic) AND TITLE-ABS-KEY ((early AND warning) OR (machine AND learning) OR prediction OR (artificial AND intelligence)) AND TITLE-ABS-KEY (mortality) AND TITLE-ABS-KEY (randomized OR controlled OR pre-implementation OR pre-post) AND (LIMIT-TO (DOCTYPE , "ar"))

WOS (n = 785)

Item number	Searching words	Number of items
1#	**((AB=(sepsis)) OR AB=(septic)) OR AB=(septicemia)**	209874
2#	(((((AB=(early warning)) OR AB=(machine learning)) OR AB=(prediction)) OR AB=(artificial intelligence)) OR AB=(detection)) OR AB=(automated)	6160275
3#	(AB=(mortality)) OR AB=(length of stay)	1225755
4#	((((AB=(randomized)) OR AB=(controlled)) OR AB=(observational)) OR AB=(pre-implementation)) OR AB=(pre-post)	18075728
5#	1# AND 2# AND 3# AND 4#	785

MedRxiv (n = 327)

(sepsis OR septic) AND (automated OR alert OR machine learning OR artificial intelligence OR early warning OR prediction) AND (mortality OR length of stay) AND (randomized OR pre-implementation OR pre-post OR controlled OR observational)

Embase under PICO framework (n = 643)

('sepsis'/exp OR 'septicemia'/exp OR 'septic shock'/exp) AND ('prediction'/exp OR 'early warning score'/exp OR 'machine learning'/exp OR 'artificial intelligence'/exp OR automated:ti,ab OR detection:ti,ab) AND ('mortality'/exp OR 'length of stay'/exp) AND ('randomized controlled trial'/exp OR 'observational study'/exp)

Cochrane Central Register of Controlled Trials (CENTRAL) (n = 801)

"sepsis" OR "septic" OR "septicemia" OR "bacteremia" in Title Abstract Keyword AND "detection" OR "machine learning" OR "early warning" OR "prediction" OR "artificial intelligence" OR "automated" in Title Abstract Keyword AND "mortality" OR "length of stay" in Title Abstract Keyword AND "randomized" OR "controlled" OR "observational" OR "pre-implementation" OR "pre-post" in Title Abstract Keyword - (Word variations have been searched)

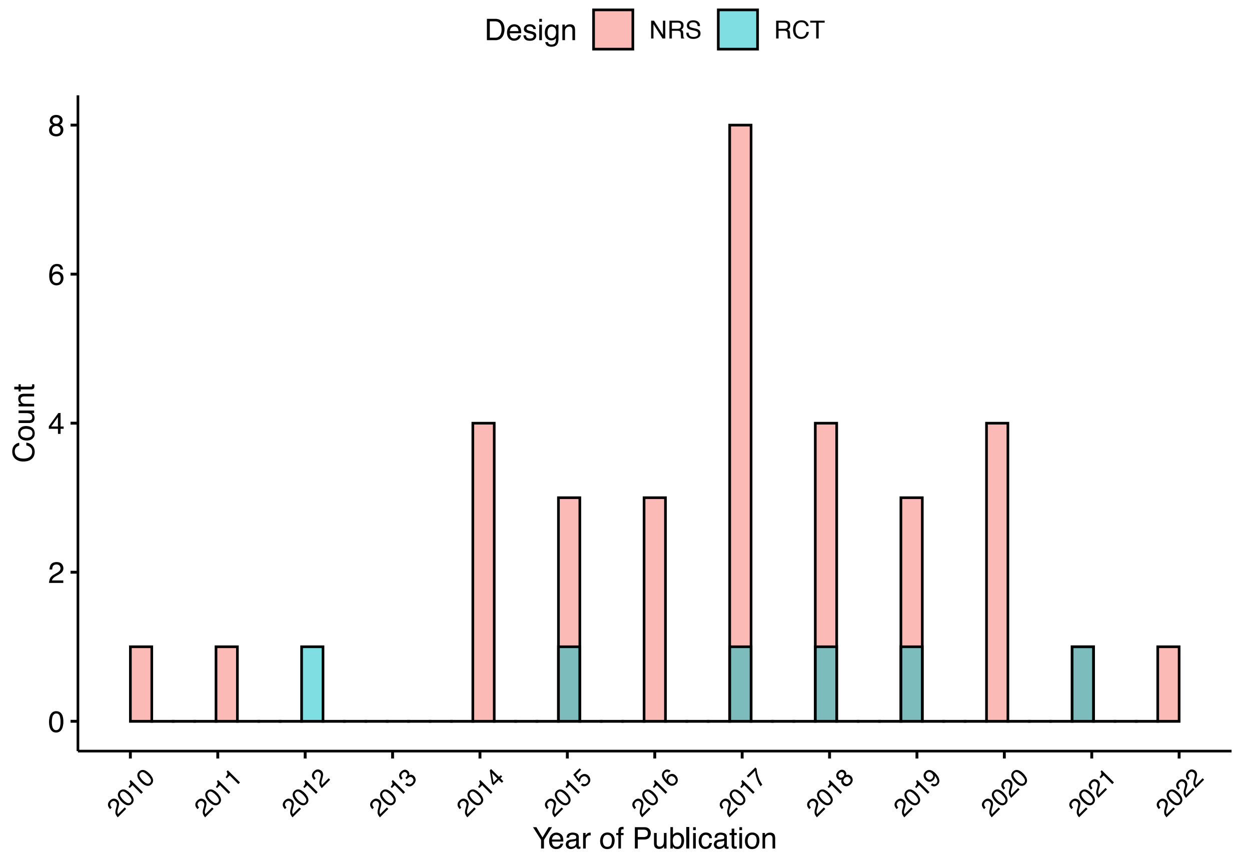

Supplementary Figure 1 Trends of the number of publications over time stratified by study design.

NRS = non-randomized study; RCT = randomized controlled trial

Study or Subgroup	Experimental Events	Experimental Total	Control Events	Control Total	Weight	Risk Ratio IV, Random, 95% CI
**Design = ICU**						
Shimabukuro 2017	6	67	16	75	1.5%	0.42 [0.17; 1.01]
Semler 2015	30	218	30	189	2.9%	0.87 [0.54; 1.38]
Hooper 2012	31	220	23	222	2.7%	1.36 [0.82; 2.26]
Idrees 2016	5	45	7	55	1.2%	0.87 [0.30; 2.57]
Croft 2014	16	119	15	65	2.2%	0.58 [0.31; 1.10]
Lipatov 2022	203	1096	166	854	4.0%	0.95 [0.79; 1.15]
**Total (95% CI)**		**1765**		**1460**	**14.5%**	**0.90 [0.73; 1.11]**
**Prediction interval**	.	.	.	.	--	**[0.57; 1.41]**
Heterogeneity: $Tau^2 = 0.0145$; $Chi^2 = 7.55$, df = 5 (P = 0.18); $I^2 = 34\%$						
**Design = ED**						
Tarabichi 2021	13	285	25	313	2.2%	0.57 [0.30; 1.09]
Gatewood 2015	14	122	18	137	2.2%	0.87 [0.45; 1.68]
Narayanan 2016	18	103	20	111	2.4%	0.97 [0.54; 1.73]
Arabi 2017a	60	195	208	436	3.8%	0.64 [0.51; 0.81]
Arabi 2017b	118	699	208	436	4.0%	0.35 [0.29; 0.43]
Berger 2010	46	890	52	908	3.2%	0.90 [0.61; 1.33]
Ferreras 2015	27	198	59	235	3.1%	0.54 [0.36; 0.82]
Hayden 2016	18	130	10	108	1.9%	1.50 [0.72; 3.10]
Machado 2018	25	95	49	219	3.1%	1.18 [0.77; 1.79]
Song 2019	93	315	118	316	3.9%	0.79 [0.63; 0.99]
Shah 2018	15	57	21	58	2.5%	0.73 [0.42; 1.26]
Threatt 2020	9	145	72	165	2.1%	0.14 [0.07; 0.27]
Honeyford 2020	339	6671	959	15056	4.2%	0.80 [0.71; 0.90]
**Total (95% CI)**		**9905**		**18498**	**38.6%**	**0.68 [0.51; 0.90]**
**Prediction interval**	.	.	.	.	--	**[0.23; 2.03]**
Heterogeneity: $Tau^2 = 0.2275$; $Chi^2 = 91.8$, df = 12 (P < 0.01); $I^2 = 87\%$						
**Design = Wards**						
Downing 2019	37	595	36	528	3.0%	0.91 [0.59; 1.42]
Downey 2018	1	140	0	86	0.2%	1.85 [0.08; 44.83]
McCoy 2017a	9	336	30	407	1.9%	0.36 [0.17; 0.75]
McCoy 2017b	12	381	30	407	2.1%	0.43 [0.22; 0.82]
McCoy 2017c	6	204	30	407	1.6%	0.40 [0.17; 0.94]
Burdick 2020	1459	62354	494	12793	4.2%	0.61 [0.55; 0.67]
Giannini 2019	220	2137	163	1540	4.0%	0.97 [0.80; 1.18]
Umscheid 2015	41	545	52	595	3.2%	0.86 [0.58; 1.27]
Austrian 2018	91	1306	71	838	3.6%	0.82 [0.61; 1.11]
Benson 2014	20	116	19	123	2.4%	1.12 [0.63; 1.98]
Guirgis 2017	202	1568	226	1637	4.0%	0.93 [0.78; 1.11]
Manaktala 2017	19	464	105	1170	2.8%	0.46 [0.28; 0.73]
Mathews 2014	392	1212	389	879	4.2%	0.73 [0.65; 0.82]
McRee 2014	1	96	7	75	0.4%	0.11 [0.01; 0.89]
Sawyer 2011	9	89	21	181	1.9%	0.87 [0.42; 1.82]
Westra 2017	33	778	518	5753	3.4%	0.47 [0.33; 0.66]
Na 2021	231	957	218	639	4.1%	0.71 [0.61; 0.83]
**Total (95% CI)**		**73278**		**28058**	**47.0%**	**0.71 [0.61; 0.82]**
**Prediction interval**	.	.	.	.	--	**[0.43; 1.16]**
Heterogeneity: $Tau^2 = 0.0493$; $Chi^2 = 54.61$, df = 16 (P < 0.01); $I^2 = 71\%$						
**Total (95% CI)**		**84948**		**48016**	**100.0%**	**0.70 [0.62; 0.81]**
**Prediction interval**						**[0.35; 1.40]**
Heterogeneity: $Tau^2 = 0.1099$; $Chi^2 = 166.87$, df = 35 (P < 0.01); $I^2 = 79\%$						
Test for subgroup differences: $Chi^2 = 3.88$, df = 2 (P = 0.14)						

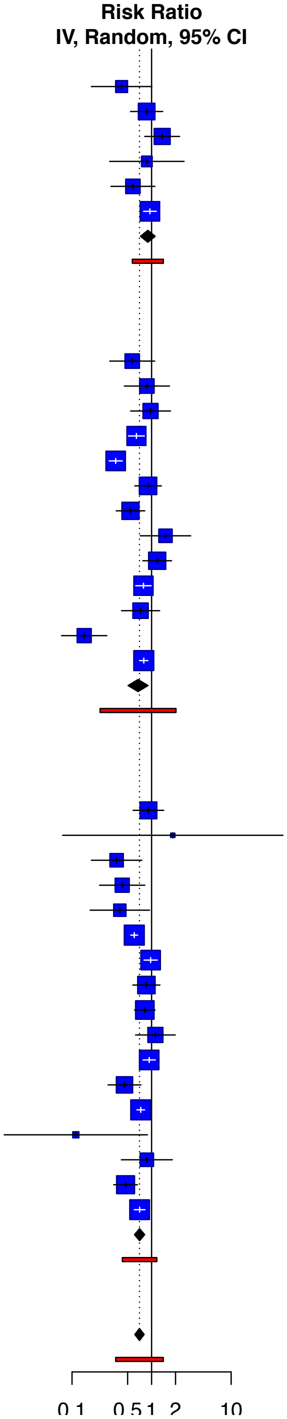

Supplementary Figure 2. Subgroup analysis for mortality outcome stratified by setting.

The size of the blue square indicates the weight of each individual study. The black diamond represents the pooled effect size for each subgroup as well as for the overall effect. The red bars represent the prediction interval.

Abbreviations: ED = emergency department; ICU = intensive care unit; CI = confidence interval; IV = inverse variance.

Study or Subgroup	Experimental Events	Experimental Total	Control Events	Control Total	Weight	Risk Ratio IV, Random, 95% CI
**Design = ML–based**						
Shimabukuro 2017	6	67	16	75	1.5%	0.42 [0.17; 1.01]
McCoy 2017a	9	336	30	407	1.9%	0.36 [0.17; 0.75]
McCoy 2017b	12	381	30	407	2.1%	0.43 [0.22; 0.82]
McCoy 2017c	6	204	30	407	1.6%	0.40 [0.17; 0.94]
Burdick 2020	1459	62354	494	12793	4.2%	0.61 [0.55; 0.67]
Giannini 2019	220	2137	163	1540	4.0%	0.97 [0.80; 1.18]
**Total (95% CI)**		**65479**		**15629**	**15.4%**	**0.56 [0.39; 0.80]**
**Prediction interval**	.	.	.	.	--	**[0.19; 1.67]**
Heterogeneity: $Tau^2 = 0.1208$; $Chi^2 = 25.05$, df = 5 ($P < 0.01$); $I^2 = 80\%$						
**Design = rule–based**						
Semler 2015	30	218	30	189	2.9%	0.87 [0.54; 1.38]
Hooper 2012	31	220	23	222	2.7%	1.36 [0.82; 2.26]
Tarabichi 2021	13	285	25	313	2.2%	0.57 [0.30; 1.09]
Downing 2019	37	595	36	528	3.0%	0.91 [0.59; 1.42]
Downey 2018	1	140	0	86	0.2%	1.85 [0.08; 44.83]
Gatewood 2015	14	122	18	137	2.2%	0.87 [0.45; 1.68]
Narayanan 2016	18	103	20	111	2.4%	0.97 [0.54; 1.73]
Umscheid 2015	41	545	52	595	3.2%	0.86 [0.58; 1.27]
Arabi 2017a	60	195	208	436	3.8%	0.64 [0.51; 0.81]
Arabi 2017b	118	699	208	436	4.0%	0.35 [0.29; 0.43]
Austrian 2018	91	1306	71	838	3.6%	0.82 [0.61; 1.11]
Benson 2014	20	116	19	123	2.4%	1.12 [0.63; 1.98]
Berger 2010	46	890	52	908	3.2%	0.90 [0.61; 1.33]
Ferreras 2015	27	198	59	235	3.1%	0.54 [0.36; 0.82]
Guirgis 2017	202	1568	226	1637	4.0%	0.93 [0.78; 1.11]
Hayden 2016	18	130	10	108	1.9%	1.50 [0.72; 3.10]
Manaktala 2017	19	464	105	1170	2.8%	0.46 [0.28; 0.73]
Mathews 2014	392	1212	389	879	4.2%	0.73 [0.65; 0.82]
McRee 2014	1	96	7	75	0.4%	0.11 [0.01; 0.89]
Sawyer 2011	9	89	21	181	1.9%	0.87 [0.42; 1.82]
Westra 2017	33	778	518	5753	3.4%	0.47 [0.33; 0.66]
Idrees 2016	5	45	7	55	1.2%	0.87 [0.30; 2.57]
Machado 2018	25	95	49	219	3.1%	1.18 [0.77; 1.79]
Song 2019	93	315	118	316	3.9%	0.79 [0.63; 0.99]
Shah 2018	15	57	21	58	2.5%	0.73 [0.42; 1.26]
Threatt 2020	9	145	72	165	2.1%	0.14 [0.07; 0.27]
Croft 2014	16	119	15	65	2.2%	0.58 [0.31; 1.10]
Lipatov 2022	203	1096	166	854	4.0%	0.95 [0.79; 1.15]
Honeyford 2020	339	6671	959	15056	4.2%	0.80 [0.71; 0.90]
Na 2021	231	957	218	639	4.1%	0.71 [0.61; 0.83]
**Total (95% CI)**		**19469**		**32387**	**84.6%**	**0.73 [0.63; 0.85]**
**Prediction interval**	.	.	.	.	--	**[0.36; 1.48]**
Heterogeneity: $Tau^2 = 0.1128$; $Chi^2 = 136.48$, df = 29 ($P < 0.01$); $I^2 = 79\%$						
**Total (95% CI)**		**84948**		**48016**	**100.0%**	**0.70 [0.62; 0.81]**
**Prediction interval**						**[0.35; 1.40]**
Heterogeneity: $Tau^2 = 0.1099$; $Chi^2 = 166.87$, df = 35 ($P < 0.01$); $I^2 = 79\%$						
Test for subgroup differences: $Chi^2 = 1.84$, df = 1 ($P = 0.18$)						

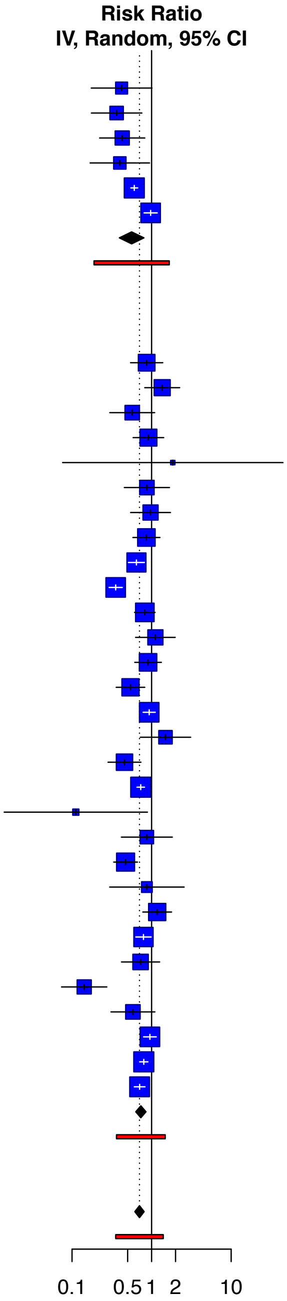

Supplementary Figure 3. Subgroup analysis for mortality outcome stratified by alerting method.

The size of the blue square indicates the weight of each individual study. The black diamond represents the pooled effect size for each subgroup as well as for the overall effect. The red bars represent the prediction interval.

Abbreviations: ED = emergency department; ICU = intensive care unit; CI = confidence interval; IV = inverse variance.

Subgroup	Events	Total	Events	Total	Weight	IV, Random, 95% CI
Design = severe sepsis alert						
Shimabukuro 2017	6	67	16	75	1.5%	0.42 [0.17; 1.01]
Downing 2019	37	595	36	528	3.0%	0.91 [0.59; 1.42]
Burdick 2020	1459	62354	494	12793	4.2%	0.61 [0.55; 0.67]
Narayanan 2016	18	103	20	111	2.4%	0.97 [0.54; 1.73]
Giannini 2019	220	2137	163	1540	4.0%	0.97 [0.80; 1.18]
Umscheid 2015	41	545	52	595	3.2%	0.86 [0.58; 1.27]
Berger 2010	46	890	52	908	3.2%	0.90 [0.61; 1.33]
Mathews 2014	392	1212	389	879	4.2%	0.73 [0.65; 0.82]
Total (95% CI)		67903		17429	25.7%	0.78 [0.66; 0.92]
Prediction interval	.	.	.	.	---	[0.49; 1.25]
Heterogeneity: $Tau^2 = 0.0296$; $Chi^2 = 26.35$, df = 7 ($P < 0.01$); $I^2 = 73\%$						
Design = bundle implementation						
Semler 2015	30	218	30	189	2.9%	0.87 [0.54; 1.38]
Arabi 2017a	60	195	208	436	3.8%	0.64 [0.51; 0.81]
Arabi 2017b	118	699	208	436	4.0%	0.35 [0.29; 0.43]
Hayden 2016	18	130	10	108	1.9%	1.50 [0.72; 3.10]
Song 2019	93	315	118	316	3.9%	0.79 [0.63; 0.99]
Threatt 2020	9	145	72	165	2.1%	0.14 [0.07; 0.27]
Croft 2014	16	119	15	65	2.2%	0.58 [0.31; 1.10]
Lipatov 2022	203	1096	166	854	4.0%	0.95 [0.79; 1.15]
Honeyford 2020	339	6671	959	15056	4.2%	0.80 [0.71; 0.90]
Total (95% CI)		9588		17625	29.0%	0.63 [0.43; 0.94]
Prediction interval	.	.	.	.	---	[0.15; 2.61]
Heterogeneity: $Tau^2 = 0.3187$; $Chi^2 = 93.48$, df = 8 ($P < 0.01$); $I^2 = 91\%$						
Design = sepsis alert						
Hooper 2012	31	220	23	222	2.7%	1.36 [0.82; 2.26]
Tarabichi 2021	13	285	25	313	2.2%	0.57 [0.30; 1.09]
Downey 2018	1	140	0	86	0.2%	1.85 [0.08; 44.83]
McCoy 2017a	9	336	30	407	1.9%	0.36 [0.17; 0.75]
McCoy 2017b	12	381	30	407	2.1%	0.43 [0.22; 0.82]
McCoy 2017c	6	204	30	407	1.6%	0.40 [0.17; 0.94]
Gatewood 2015	14	122	18	137	2.2%	0.87 [0.45; 1.68]
Austrian 2018	91	1306	71	838	3.6%	0.82 [0.61; 1.11]
Benson 2014	20	116	19	123	2.4%	1.12 [0.63; 1.98]
Ferreras 2015	27	198	59	235	3.1%	0.54 [0.36; 0.82]
Guirgis 2017	202	1568	226	1637	4.0%	0.93 [0.78; 1.11]
Manaktala 2017	19	464	105	1170	2.8%	0.46 [0.28; 0.73]
McRee 2014	1	96	7	75	0.4%	0.11 [0.01; 0.89]
Sawyer 2011	9	89	21	181	1.9%	0.87 [0.42; 1.82]
Westra 2017	33	778	518	5753	3.4%	0.47 [0.33; 0.66]
Idrees 2016	5	45	7	55	1.2%	0.87 [0.30; 2.57]
Machado 2018	25	95	49	219	3.1%	1.18 [0.77; 1.79]
Shah 2018	15	57	21	58	2.5%	0.73 [0.42; 1.26]
Na 2021	231	957	218	639	4.1%	0.71 [0.61; 0.83]
Total (95% CI)		7457		12962	45.3%	0.70 [0.59; 0.85]
Prediction interval	.	.	.	.	---	[0.37; 1.33]
Heterogeneity: $Tau^2 = 0.0830$; $Chi^2 = 45.29$, df = 18 ($P < 0.01$); $I^2 = 60\%$						
**Total (95% CI)**		**84948**		**48016**	**100.0%**	**0.70 [0.62; 0.81]**
**Prediction interval**						**[0.35; 1.40]**
Heterogeneity: $Tau^2 = 0.1099$; $Chi^2 = 166.87$, df = 35 ($P < 0.01$); $I^2 = 79\%$						

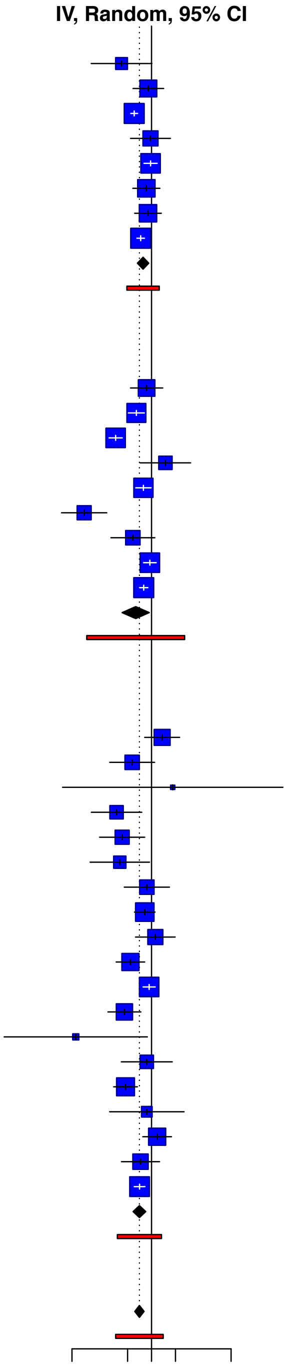

Supplementary Figure 4. Subgroup analysis for mortality outcome stratified by purpose of alerting.

The size of the blue square indicates the weight of each individual study. The black diamond represents the pooled effect size for each subgroup as well as for the overall effect. The red bars represent the prediction interval.

Abbreviations: ED = emergency department; ICU = intensive care unit; CI = confidence interval; IV = inverse variance.

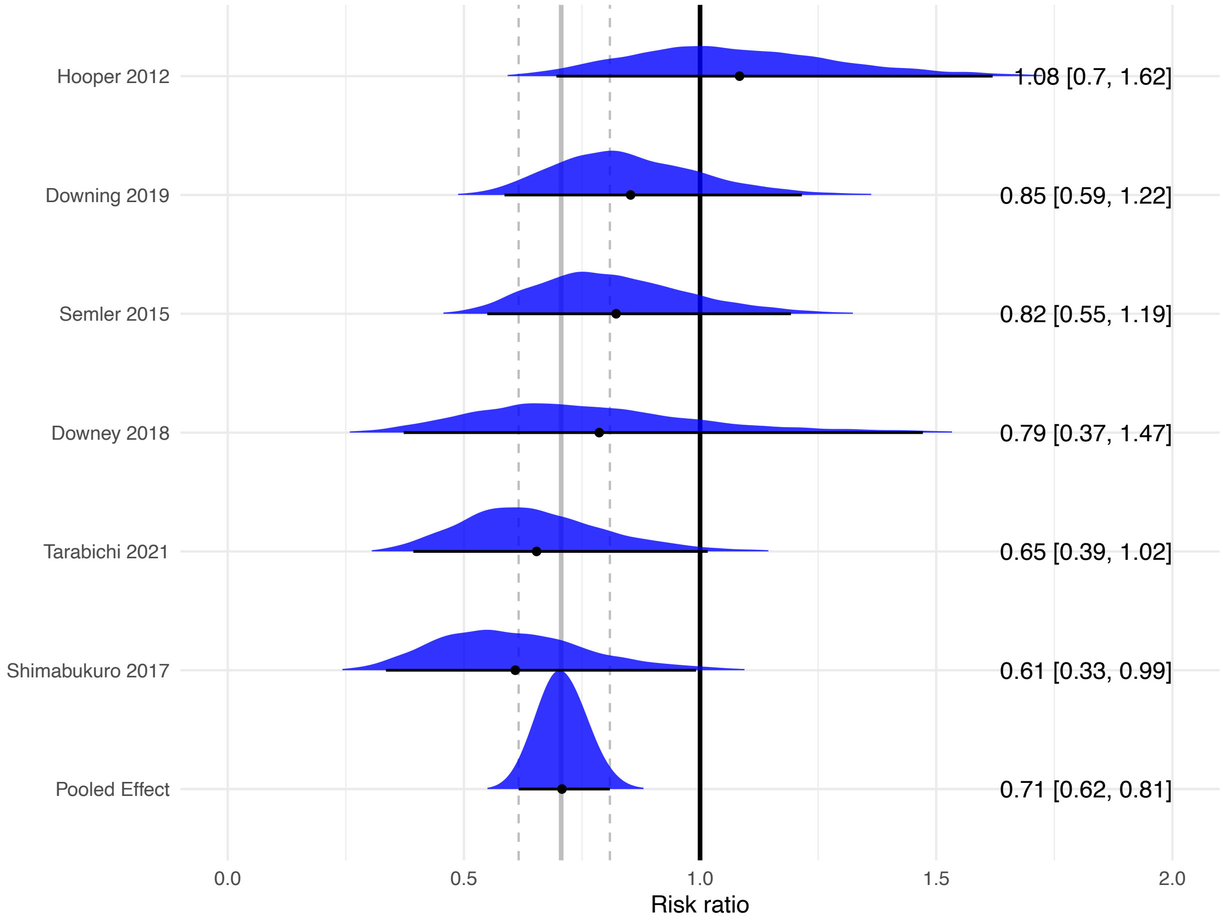

Supplementary Figure 5. Bayesian meta-analysis of RCTs for mortality.
Pooled metrics from non-randomized studies were employed as the prior. The effect measure is the risk ratio, and a value <1 indicates beneficial effect of the automated alerting system. The blue shaded area represents the posterior distribution for each individual study as well as for the pooled effects.

Study or Subgroup	Experimental Mean	Experimental SD	Experimental Total	Control Mean	Control SD	Control Total	Weight	Mean Difference IV, Random, 95% CI
**Design = RCT**								
Shimabukuro 2017	6.31	0.6660	67	8.40	0.8810	75	9.4%	−2.09 [−2.35; −1.83]
Hooper 2012	3.35	2.2387	220	3.00	1.4924	222	9.3%	0.35 [0.00; 0.71]
Tarabichi 2021	3.67	2.5334	285	3.82	2.9791	313	9.3%	−0.15 [−0.59; 0.29]
**Total (95% CI)**			**572**			**610**	**28.0%**	**−0.64 [−2.10; 0.83]**
**Prediction interval**			.			.	**--**	**[−19.52; 18.24]**
Heterogeneity: $Tau^2 = 1.6474$; $Chi^2 = 139.44$, df = 2 ($P < 0.01$); $I^2 = 99\%$								
**Design = NRS**								
Giannini 2019	4.30	4.2036	2137	3.82	3.8646	1540	9.4%	0.48 [0.22; 0.74]
Umscheid 2015	4.13	3.6421	545	4.21	4.2358	595	9.3%	−0.08 [−0.54; 0.38]
Austrian 2018	1.20	3.1000	1306	1.80	3.7000	838	9.3%	−0.60 [−0.90; −0.30]
Guirgis 2017	1.05	2.2260	1568	1.75	3.7098	1637	9.4%	−0.70 [−0.91; −0.49]
Hayden 2016	6.20	8.4000	130	4.10	2.9000	108	8.9%	2.10 [0.56; 3.64]
Shah 2018	3.00	4.5593	57	6.65	9.4841	58	8.0%	−3.65 [−6.36; −0.94]
Jung 2018	7.60	2.5000	7	19.10	3.3000	23	8.4%	−11.50 [−13.79; −9.21]
Lipatov 2022	2.88	2.2269	1096	2.96	2.3020	854	9.4%	−0.08 [−0.28; 0.13]
**Total (95% CI)**			**6846**			**5653**	**72.0%**	**−1.65 [−4.48; 1.18]**
**Prediction interval**			.			.	**--**	**[−12.12; 8.82]**
Heterogeneity: $Tau^2 = 16.2113$; $Chi^2 = 163.39$, df = 7 ($P < 0.01$); $I^2 = 96\%$								
**Total (95% CI)**			**7418**			**6263**	**100.0%**	**−1.33 [−3.34; 0.67]**
**Prediction interval**								**[−9.26; 6.59]**
Heterogeneity: $Tau^2 = 11.2109$; $Chi^2 = 355.12$, df = 10 ($P < 0.01$); $I^2 = 97\%$								
Test for subgroup differences: $Chi^2 = 0.39$, df = 1 ($P = 0.53$)								

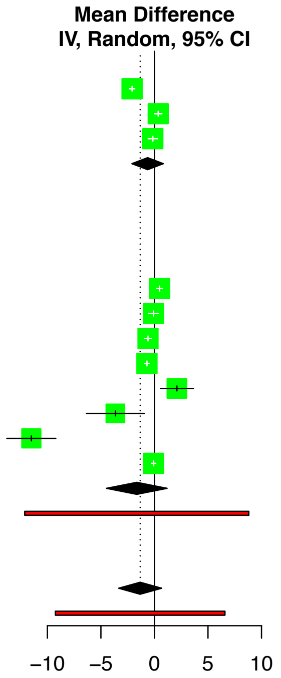

Supplementary Figure 6. Subgroup analysis for ICU length of stay stratified by the study design.

The size of the green square indicates the weight of each individual study. The black diamond represents the pooled effect size for each subgroup as well as for the overall effect. The red bars represent the prediction interval.

Abbreviations: RCT = randomized controlled trial; ICU = intensive care unit; CI = confidence interval; IV = inverse variance.

Study or Subgroup	Experimental Mean	Experimental SD	Experimental Total	Control Mean	Control SD	Control Total	Weight	Mean Difference IV, Random, 95% CI
**Design = ICU**								
Shimabukuro 2017	6.31	0.6660	67	8.40	0.8810	75	9.4%	−2.09 [−2.35; −1.83]
Hooper 2012	3.35	2.2387	220	3.00	1.4924	222	9.3%	0.35 [0.00; 0.71]
Jung 2018	7.60	2.5000	7	19.10	3.3000	23	8.4%	−11.50 [−13.79; −9.21]
Lipatov 2022	2.88	2.2269	1096	2.96	2.3020	854	9.4%	−0.08 [−0.28; 0.13]
**Total (95% CI)**			**1390**			**1174**	**36.4%**	**−3.24 [−8.56; 2.09]**
**Prediction interval**			.			.	**--**	**[−29.25; 22.77]**
Heterogeneity: $Tau^2 = 29.1619$; $Chi^2 = 269.27$, df = 3 ($P < 0.01$); $I^2 = 99\%$								
**Design = ED**								
Tarabichi 2021	3.67	2.5334	285	3.82	2.9791	313	9.3%	−0.15 [−0.59; 0.29]
Hayden 2016	6.20	8.4000	130	4.10	2.9000	108	8.9%	2.10 [0.56; 3.64]
Shah 2018	3.00	4.5593	57	6.65	9.4841	58	8.0%	−3.65 [−6.36; −0.94]
**Total (95% CI)**			**472**			**479**	**26.2%**	**−0.39 [−3.45; 2.66]**
**Prediction interval**			.			.	**--**	**[−38.41; 37.62]**
Heterogeneity: $Tau^2 = 6.5174$; $Chi^2 = 14.41$, df = 2 ($P < 0.01$); $I^2 = 86\%$								
**Design = Wards**								
Giannini 2019	4.30	4.2036	2137	3.82	3.8646	1540	9.4%	0.48 [0.22; 0.74]
Umscheid 2015	4.13	3.6421	545	4.21	4.2358	595	9.3%	−0.08 [−0.54; 0.38]
Austrian 2018	1.20	3.1000	1306	1.80	3.7000	838	9.3%	−0.60 [−0.90; −0.30]
Guirgis 2017	1.05	2.2260	1568	1.75	3.7098	1637	9.4%	−0.70 [−0.91; −0.49]
**Total (95% CI)**			**5556**			**4610**	**37.4%**	**−0.23 [−0.78; 0.32]**
**Prediction interval**			.			.	**--**	**[−2.82; 2.36]**
Heterogeneity: $Tau^2 = 0.2856$; $Chi^2 = 52.49$, df = 3 ($P < 0.01$); $I^2 = 94\%$								
**Total (95% CI)**			**7418**			**6263**	**100.0%**	**−1.33 [−3.34; 0.67]**
**Prediction interval**								**[−9.26; 6.59]**
Heterogeneity: $Tau^2 = 11.2109$; $Chi^2 = 355.12$, df = 10 ($P < 0.01$); $I^2 = 97\%$								
Test for subgroup differences: $Chi^2 = 1.22$, df = 2 ($P = 0.54$)								

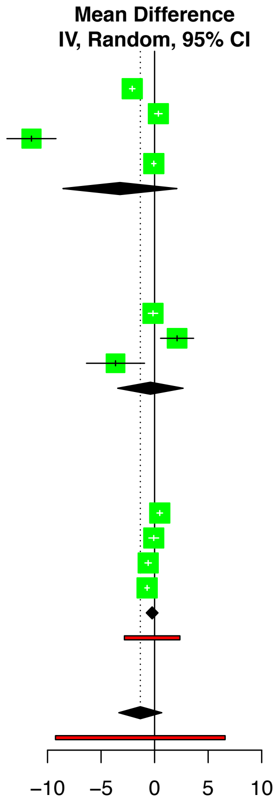

Supplementary Figure 7. Subgroup analysis for ICU length of stay stratified by the study setting.

The size of the green square indicates the weight of each individual study. The black diamond represents the pooled effect size for each subgroup as well as for the overall effect. The red bars represent the prediction interval.

Abbreviations: ED = emergency department; ICU = intensive care unit; CI = confidence interval; IV = inverse variance.

Study or Subgroup	Experimental Mean	Experimental SD	Experimental Total	Control Mean	Control SD	Control Total	Weight	Mean Difference IV, Random, 95% CI
Design = ML–based								
Shimabukuro 2017	6.31	0.6660	67	8.40	0.8810	75	9.4%	−2.09 [−2.35; −1.83]
Giannini 2019	4.30	4.2036	2137	3.82	3.8646	1540	9.4%	0.48 [0.22; 0.74]
Total (95% CI)			2204			1615	18.7%	−0.80 [−3.32; 1.72]
Prediction interval			.			.		--
Heterogeneity: Tau^2 = 3.2879; Chi^2 = 189.23, df = 1 (P < 0.01); I^2 = 99%								
Design = rule–based								
Hooper 2012	3.35	2.2387	220	3.00	1.4924	222	9.3%	0.35 [0.00; 0.71]
Tarabichi 2021	3.67	2.5334	285	3.82	2.9791	313	9.3%	−0.15 [−0.59; 0.29]
Umscheid 2015	4.13	3.6421	545	4.21	4.2358	595	9.3%	−0.08 [−0.54; 0.38]
Austrian 2018	1.20	3.1000	1306	1.80	3.7000	838	9.3%	−0.60 [−0.90; −0.30]
Guirgis 2017	1.05	2.2260	1568	1.75	3.7098	1637	9.4%	−0.70 [−0.91; −0.49]
Hayden 2016	6.20	8.4000	130	4.10	2.9000	108	8.9%	2.10 [0.56; 3.64]
Shah 2018	3.00	4.5593	57	6.65	9.4841	58	8.0%	−3.65 [−6.36; −0.94]
Jung 2018	7.60	2.5000	7	19.10	3.3000	23	8.4%	−11.50 [−13.79; −9.21]
Lipatov 2022	2.88	2.2269	1096	2.96	2.3020	854	9.4%	−0.08 [−0.28; 0.13]
Total (95% CI)			5214			4648	81.3%	−1.48 [−3.97; 1.01]
Prediction interval			.			.		-- [−10.84; 7.88]
Heterogeneity: Tau^2 = 14.0653; Chi^2 = 143.44, df = 8 (P < 0.01); I^2 = 94%								
**Total (95% CI)**			**7418**			**6263**	**100.0%**	**−1.33 [−3.34; 0.67]**
**Prediction interval**								**[−9.26; 6.59]**
Heterogeneity: Tau^2 = 11.2109; Chi^2 = 355.12, df = 10 (P < 0.01); I^2 = 97%								
Test for subgroup differences: Chi^2 = 0.14, df = 1 (P = 0.71)								

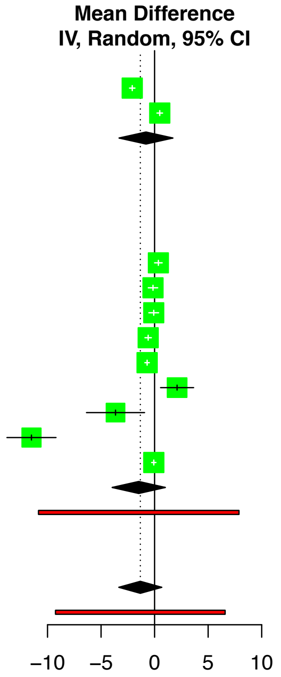

Supplementary Figure 8. Subgroup analysis for ICU length of stay stratified by prediction methods.

The size of the green square indicates the weight of each individual study. The black diamond represents the pooled effect size for each subgroup as well as for the overall effect. The red bars represent the prediction interval.

Abbreviations: ML = machine learning; ICU = intensive care unit; CI = confidence interval; IV = inverse variance.

Study or Subgroup	Experimental Mean	SD	Total	Control Mean	SD	Total	Weight	Mean Difference IV, Random, 95% CI
**Design = severe sepsis alert**								
Shimabukuro 2017	6.31	0.6660	67	8.40	0.8810	75	9.4%	−2.09 [−2.35; −1.83]
Giannini 2019	4.30	4.2036	2137	3.82	3.8646	1540	9.4%	0.48 [0.22; 0.74]
Umscheid 2015	4.13	3.6421	545	4.21	4.2358	595	9.3%	−0.08 [−0.54; 0.38]
**Total (95% CI)**			**2749**			**2210**	**28.0%**	**−0.57 [−2.10; 0.97]**
**Prediction interval**			.			.	**--**	**[−20.39; 19.26]**
Heterogeneity: Tau^2 = 1.8179; Chi^2 = 198.38, df = 2 (P < 0.01); I^2 = 99%								
**Design = sepsis alert**								
Hooper 2012	3.35	2.2387	220	3.00	1.4924	222	9.3%	0.35 [0.00; 0.71]
Tarabichi 2021	3.67	2.5334	285	3.82	2.9791	313	9.3%	−0.15 [−0.59; 0.29]
Austrian 2018	1.20	3.1000	1306	1.80	3.7000	838	9.3%	−0.60 [−0.90; −0.30]
Guirgis 2017	1.05	2.2260	1568	1.75	3.7098	1637	9.4%	−0.70 [−0.91; −0.49]
Shah 2018	3.00	4.5593	57	6.65	9.4841	58	8.0%	−3.65 [−6.36; −0.94]
Jung 2018	7.60	2.5000	7	19.10	3.3000	23	8.4%	−11.50 [−13.79; −9.21]
**Total (95% CI)**			**3443**			**3091**	**53.7%**	**−2.59 [−6.13; 0.94]**
**Prediction interval**			.			.	**--**	**[−15.67; 10.48]**
Heterogeneity: Tau^2 = 18.9377; Chi^2 = 122.08, df = 5 (P < 0.01); I^2 = 96%								
**Design = bundle implementation**								
Hayden 2016	6.20	8.4000	130	4.10	2.9000	108	8.9%	2.10 [0.56; 3.64]
Lipatov 2022	2.88	2.2269	1096	2.96	2.3020	854	9.4%	−0.08 [−0.28; 0.13]
**Total (95% CI)**			**1226**			**962**	**18.2%**	**0.87 [−1.24; 2.99]**
**Prediction interval**			.			.	**--**	
Heterogeneity: Tau^2 = 2.0500; Chi^2 = 7.49, df = 1 (P < 0.01); I^2 = 87%								
**Total (95% CI)**			**7418**			**6263**	**100.0%**	**−1.33 [−3.34; 0.67]**
**Prediction interval**								**[−9.26; 6.59]**
Heterogeneity: Tau^2 = 11.2109; Chi^2 = 355.12, df = 10 (P < 0.01); I^2 = 97%								
Test for subgroup differences: Chi^2 = 2.91, df = 2 (P = 0.23)								

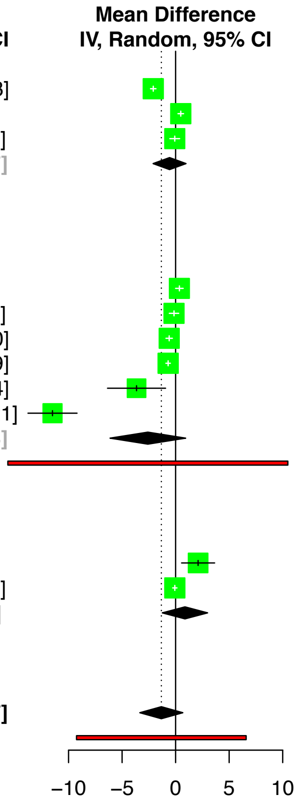

Supplementary Figure 9. Subgroup analysis for ICU length of stay stratified by prediction purpose.

The size of the green square indicates the weight of each individual study. The black diamond represents the pooled effect size for each subgroup as well as for the overall effect. The red bars represent the prediction interval.

Abbreviations: SD = standard deviation; ICU = intensive care unit; CI = confidence interval; IV = inverse variance.

Study or Subgroup	Experimental Mean	SD	Total	Control Mean	SD	Total	Weight	Mean Difference IV, Random, 95% CI
**Design = RCT**								
Shimabukuro 2017	10.30	0.9120	67	13.00	1.2300	75	5.0%	−2.70 [−3.05; −2.35]
Hooper 2012	6.37	5.7460	220	5.19	4.0294	222	5.0%	1.18 [0.25; 2.10]
Tarabichi 2021	3.52	3.8000	285	4.14	4.1707	313	5.0%	−0.62 [−1.26; 0.01]
Downey 2018	13.30	1.0200	140	14.60	1.5300	86	5.0%	−1.30 [−1.66; −0.94]
**Total (95% CI)**			**712**			**696**	**20.1%**	**−0.90 [−2.46; 0.65]**
**Prediction interval**			.			.	--	**[−8.42; 6.62]**
Heterogeneity: Tau^2 = 2.4242; Chi^2 = 83.03, df = 3 (P < 0.01); I^2 = 96%								
**Design = NRS**								
Giannini 2019	10.75	9.6435	2137	10.75	9.6460	1540	5.0%	−0.00 [−0.63; 0.63]
Umscheid 2015	11.26	10.1830	545	11.50	10.4037	595	5.0%	−0.24 [−1.44; 0.95]
Arabi 2017a	25.70	40.9000	195	29.30	33.9000	436	3.3%	−3.60 [−10.16; 2.96]
Arabi 2017b	15.30	23.5000	699	29.30	33.9000	436	4.3%	−14.00 [−17.63; −10.37]
Austrian 2018	8.60	7.9000	1306	10.10	10.1000	838	5.0%	−1.50 [−2.31; −0.69]
Guirgis 2017	8.75	8.1619	1568	9.75	9.6455	1637	5.0%	−1.00 [−1.62; −0.38]
Hayden 2016	9.00	10.1000	130	8.20	9.2000	108	4.7%	0.80 [−1.65; 3.25]
McRee 2014	8.70	6.0000	96	8.50	6.0000	75	4.8%	0.20 [−1.61; 2.01]
Sawyer 2011	9.70	7.5301	89	8.76	6.7214	181	4.8%	0.95 [−0.90; 2.79]
Idrees 2016	7.71	4.5907	45	10.12	9.1185	55	4.6%	−2.41 [−5.17; 0.35]
Machado 2018	8.60	5.7000	95	10.00	8.5000	219	4.9%	−1.40 [−3.01; 0.21]
Shah 2018	8.12	7.5988	57	9.41	9.1047	58	4.5%	−1.29 [−4.36; 1.77]
Moore 2019	4.50	4.4800	202	7.07	8.9900	110	4.9%	−2.57 [−4.36; −0.78]
Threatt 2020	9.17	8.9700	145	9.15	10.7700	165	4.8%	0.02 [−2.18; 2.22]
Jung 2018	10.80	3.1000	7	29.60	4.3000	23	4.6%	−18.80 [−21.69; −15.91]
Lipatov 2022	8.21	6.0868	1096	8.59	6.6089	854	5.0%	−0.38 [−0.95; 0.20]
Na 2021	27.10	25.2427	957	32.15	27.4908	639	4.6%	−5.05 [−7.72; −2.39]
**Total (95% CI)**			**9369**			**7969**	**79.9%**	**−2.83 [−5.32; −0.35]**
**Prediction interval**			.			.	--	**[−14.00; 8.33]**
Heterogeneity: Tau^2 = 25.8396; Chi^2 = 234.37, df = 16 (P < 0.01); I^2 = 93%								
**Total (95% CI)**			**10081**			**8665**	**100.0%**	**−2.42 [−4.43; −0.41]**
**Prediction interval**								**[−12.21; 7.37]**
Heterogeneity: Tau^2 = 20.8326; Chi^2 = 332.92, df = 20 (P < 0.01); I^2 = 94%								
Test for subgroup differences: Chi^2 = 1.67, df = 1 (P = 0.20)								

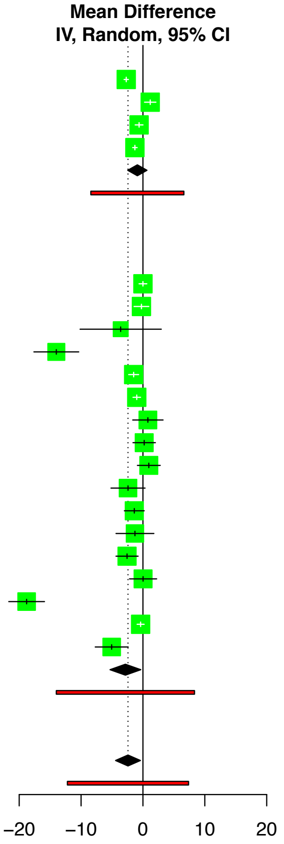

Supplementary Figure 10. Subgroup analysis for hospital length of stay stratified by study design.

The size of the green square indicates the weight of each individual study. The black diamond represents the pooled effect size for each subgroup as well as for the overall effect. The red bars represent the prediction interval.

Abbreviations: SD = standard deviation; RCT = randomized controlled trial; CI = confidence interval; IV = inverse variance.

Study or Subgroup	Experimental Mean	Experimental SD	Experimental Total	Control Mean	Control SD	Control Total	Weight	Mean Difference IV, Random, 95% CI
Design = ICU								
Shimabukuro 2017	10.30	0.9120	67	13.00	1.2300	75	5.0%	−2.70 [−3.05; −2.35]
Hooper 2012	6.37	5.7460	220	5.19	4.0294	222	5.0%	1.18 [0.25; 2.10]
Idrees 2016	7.71	4.5907	45	10.12	9.1185	55	4.6%	−2.41 [−5.17; 0.35]
Jung 2018	10.80	3.1000	7	29.60	4.3000	23	4.6%	−18.80 [−21.69; −15.91]
Lipatov 2022	8.21	6.0868	1096	8.59	6.6089	854	5.0%	−0.38 [−0.95; 0.20]
Total (95% CI)			1435			1229	24.2%	−4.55 [−11.53; 2.44]
Prediction interval			.			.	––	[−32.18; 23.09]
Heterogeneity: $Tau^2 = 62.7020$; $Chi^2 = 220.71$, df = 4 ($P < 0.01$); $I^2 = 98\%$								
Design = ED								
Tarabichi 2021	3.52	3.8000	285	4.14	4.1707	313	5.0%	−0.62 [−1.26; 0.01]
Arabi 2017a	25.70	40.9000	195	29.30	33.9000	436	3.3%	−3.60 [−10.16; 2.96]
Arabi 2017b	15.30	23.5000	699	29.30	33.9000	436	4.3%	−14.00 [−17.63; −10.37]
Hayden 2016	9.00	10.1000	130	8.20	9.2000	108	4.7%	0.80 [−1.65; 3.25]
Machado 2018	8.60	5.7000	95	10.00	8.5000	219	4.9%	−1.40 [−3.01; 0.21]
Shah 2018	8.12	7.5988	57	9.41	9.1047	58	4.5%	−1.29 [−4.36; 1.77]
Moore 2019	4.50	4.4800	202	7.07	8.9900	110	4.9%	−2.57 [−4.36; −0.78]
Threatt 2020	9.17	8.9700	145	9.15	10.7700	165	4.8%	0.02 [−2.18; 2.22]
Total (95% CI)			1808			1845	36.4%	−2.65 [−5.81; 0.51]
Prediction interval			.			.	––	[−13.91; 8.61]
Heterogeneity: $Tau^2 = 18.5609$; $Chi^2 = 57.29$, df = 7 ($P < 0.01$); $I^2 = 88\%$								
Design = Wards								
Downey 2018	13.30	1.0200	140	14.60	1.5300	86	5.0%	−1.30 [−1.66; −0.94]
Giannini 2019	10.75	9.6435	2137	10.75	9.6460	1540	5.0%	−0.00 [−0.63; 0.63]
Umscheid 2015	11.26	10.1830	545	11.50	10.4037	595	5.0%	−0.24 [−1.44; 0.95]
Austrian 2018	8.60	7.9000	1306	10.10	10.1000	838	5.0%	−1.50 [−2.31; −0.69]
Guirgis 2017	8.75	8.1619	1568	9.75	9.6455	1637	5.0%	−1.00 [−1.62; −0.38]
McRee 2014	8.70	6.0000	96	8.50	6.0000	75	4.8%	0.20 [−1.61; 2.01]
Sawyer 2011	9.70	7.5301	89	8.76	6.7214	181	4.8%	0.95 [−0.90; 2.79]
Na 2021	27.10	25.2427	957	32.15	27.4908	639	4.6%	−5.05 [−7.72; −2.39]
Total (95% CI)			6838			5591	39.4%	−0.82 [−1.58; −0.07]
Prediction interval			.			.	––	[−3.22; 1.58]
Heterogeneity: $Tau^2 = 0.8162$; $Chi^2 = 30.04$, df = 7 ($P < 0.01$); $I^2 = 77\%$								
**Total (95% CI)**			**10081**			**8665**	**100.0%**	**−2.42 [−4.43; −0.41]**
**Prediction interval**								**[−12.21; 7.37]**

Heterogeneity: $Tau^2 = 20.8326$; $Chi^2 = 332.92$, df = 20 ($P < 0.01$); $I^2 = 94\%$
Test for subgroup differences: $Chi^2 = 2.24$, df = 2 ($P = 0.33$)

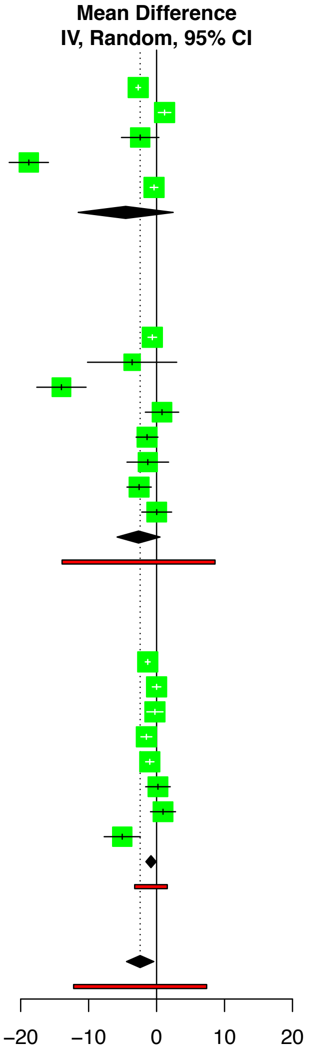

Supplementary Figure 11. Subgroup analysis for hospital length of stay stratified by study setting.
The size of the green square indicates the weight of each individual study. The black diamond represents the pooled effect size for each subgroup as well as for the overall effect. The red bars represent the prediction interval.

Abbreviations: SD = standard deviation; ED = emergency department; ICU = intensive care unit; CI = confidence interval; IV = inverse variance.

Study or Subgroup	Experimental Mean	SD	Total	Control Mean	SD	Total	Weight	Mean Difference IV, Random, 95% CI
**Design = ML–based**								
Shimabukuro 2017	10.30	0.9120	67	13.00	1.2300	75	5.0%	−2.70 [−3.05; −2.35]
Giannini 2019	10.75	9.6435	2137	10.75	9.6460	1540	5.0%	−0.00 [−0.63; 0.63]
**Total (95% CI)**			2204			1615	10.1%	−1.36 [−4.01; 1.28]
**Prediction interval**			.			.	––	
Heterogeneity: $Tau^2 = 3.5763$; $Chi^2 = 53.39$, df = 1 ($P < 0.01$); $I^2 = 98\%$								
**Design = rule–based**								
Hooper 2012	6.37	5.7460	220	5.19	4.0294	222	5.0%	1.18 [0.25; 2.10]
Tarabichi 2021	3.52	3.8000	285	4.14	4.1707	313	5.0%	−0.62 [−1.26; 0.01]
Downey 2018	13.30	1.0200	140	14.60	1.5300	86	5.0%	−1.30 [−1.66; −0.94]
Umscheid 2015	11.26	10.1830	545	11.50	10.4037	595	5.0%	−0.24 [−1.44; 0.95]
Arabi 2017a	25.70	40.9000	195	29.30	33.9000	436	3.3%	−3.60 [−10.16; 2.96]
Arabi 2017b	15.30	23.5000	699	29.30	33.9000	436	4.3%	−14.00 [−17.63; −10.37]
Austrian 2018	8.60	7.9000	1306	10.10	10.1000	838	5.0%	−1.50 [−2.31; −0.69]
Guirgis 2017	8.75	8.1619	1568	9.75	9.6455	1637	5.0%	−1.00 [−1.62; −0.38]
Hayden 2016	9.00	10.1000	130	8.20	9.2000	108	4.7%	0.80 [−1.65; 3.25]
McRee 2014	8.70	6.0000	96	8.50	6.0000	75	4.8%	0.20 [−1.61; 2.01]
Sawyer 2011	9.70	7.5301	89	8.76	6.7214	181	4.8%	0.95 [−0.90; 2.79]
Idrees 2016	7.71	4.5907	45	10.12	9.1185	55	4.6%	−2.41 [−5.17; 0.35]
Machado 2018	8.60	5.7000	95	10.00	8.5000	219	4.9%	−1.40 [−3.01; 0.21]
Shah 2018	8.12	7.5988	57	9.41	9.1047	58	4.5%	−1.29 [−4.36; 1.77]
Moore 2019	4.50	4.4800	202	7.07	8.9900	110	4.9%	−2.57 [−4.36; −0.78]
Threatt 2020	9.17	8.9700	145	9.15	10.7700	165	4.8%	0.02 [−2.18; 2.22]
Jung 2018	10.80	3.1000	7	29.60	4.3000	23	4.6%	−18.80 [−21.69; −15.91]
Lipatov 2022	8.21	6.0868	1096	8.59	6.6089	854	5.0%	−0.38 [−0.95; 0.20]
Na 2021	27.10	25.2427	957	32.15	27.4908	639	4.6%	−5.05 [−7.72; −2.39]
**Total (95% CI)**			7877			7050	89.9%	−2.55 [−4.79; −0.32]
**Prediction interval**			.			.	––	[−13.01; 7.91]
Heterogeneity: $Tau^2 = 23.2928$; $Chi^2 = 250.18$, df = 18 ($P < 0.01$); $I^2 = 93\%$								
**Total (95% CI)**			10081			8665	100.0%	−2.42 [−4.43; −0.41]
**Prediction interval**								[−12.21; 7.37]
Heterogeneity: $Tau^2 = 20.8326$; $Chi^2 = 332.92$, df = 20 ($P < 0.01$); $I^2 = 94\%$								
Test for subgroup differences: $Chi^2 = 0.45$, df = 1 ($P = 0.50$)								

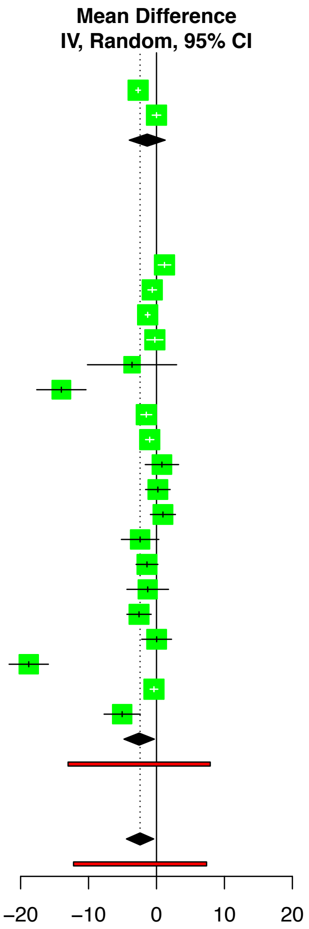

Supplementary Figure 12. Subgroup analysis for hospital length of stay stratified by alerting methods.

The size of the green square indicates the weight of each individual study. The black diamond represents the pooled effect size for each subgroup as well as for the overall effect. The red bars represent the prediction interval.

Abbreviations: SD = standard deviation; ML = machine learning; CI = confidence interval; IV = inverse variance.

Study or Subgroup	Experimental Mean	SD	Total	Control Mean	SD	Total	Weight	Mean Difference IV, Random, 95% CI
**Design = severe sepsis alert**								
Shimabukuro 2017	10.30	0.9120	67	13.00	1.2300	75	5.0%	−2.70 [−3.05; −2.35]
Giannini 2019	10.75	9.6435	2137	10.75	9.6460	1540	5.0%	−0.00 [−0.63; 0.63]
Umscheid 2015	11.26	10.1830	545	11.50	10.4037	595	5.0%	−0.24 [−1.44; 0.95]
**Total (95% CI)**			**2749**			**2210**	**15.0%**	**−1.03 [−2.77; 0.72]**
**Prediction interval**			.			.	**--**	**[−23.08; 21.02]**
Heterogeneity: $Tau^2 = 2.2186$; $Chi^2 = 61.65$, df = 2 ($P < 0.01$); $I^2 = 97\%$								
**Design = sepsis alert**								
Hooper 2012	6.37	5.7460	220	5.19	4.0294	222	5.0%	1.18 [0.25; 2.10]
Tarabichi 2021	3.52	3.8000	285	4.14	4.1707	313	5.0%	−0.62 [−1.26; 0.01]
Downey 2018	13.30	1.0200	140	14.60	1.5300	86	5.0%	−1.30 [−1.66; −0.94]
Austrian 2018	8.60	7.9000	1306	10.10	10.1000	838	5.0%	−1.50 [−2.31; −0.69]
Guirgis 2017	8.75	8.1619	1568	9.75	9.6455	1637	5.0%	−1.00 [−1.62; −0.38]
McRee 2014	8.70	6.0000	96	8.50	6.0000	75	4.8%	0.20 [−1.61; 2.01]
Sawyer 2011	9.70	7.5301	89	8.76	6.7214	181	4.8%	0.95 [−0.90; 2.79]
Idrees 2016	7.71	4.5907	45	10.12	9.1185	55	4.6%	−2.41 [−5.17; 0.35]
Machado 2018	8.60	5.7000	95	10.00	8.5000	219	4.9%	−1.40 [−3.01; 0.21]
Shah 2018	8.12	7.5988	57	9.41	9.1047	58	4.5%	−1.29 [−4.36; 1.77]
Jung 2018	10.80	3.1000	7	29.60	4.3000	23	4.6%	−18.80 [−21.69; −15.91]
Na 2021	27.10	25.2427	957	32.15	27.4908	639	4.6%	−5.05 [−7.72; −2.39]
**Total (95% CI)**			**4865**			**4346**	**58.0%**	**−2.50 [−5.42; 0.42]**
**Prediction interval**			.			.	**--**	**[−14.28; 9.28]**
Heterogeneity: $Tau^2 = 25.7438$; $Chi^2 = 187.62$, df = 11 ($P < 0.01$); $I^2 = 94\%$								
**Design = bundle implementation**								
Arabi 2017a	25.70	40.9000	195	29.30	33.9000	436	3.3%	−3.60 [−10.16; 2.96]
Arabi 2017b	15.30	23.5000	699	29.30	33.9000	436	4.3%	−14.00 [−17.63; −10.37]
Hayden 2016	9.00	10.1000	130	8.20	9.2000	108	4.7%	0.80 [−1.65; 3.25]
Moore 2019	4.50	4.4800	202	7.07	8.9900	110	4.9%	−2.57 [−4.36; −0.78]
Threatt 2020	9.17	8.9700	145	9.15	10.7700	165	4.8%	0.02 [−2.18; 2.22]
Lipatov 2022	8.21	6.0868	1096	8.59	6.6089	854	5.0%	−0.38 [−0.95; 0.20]
**Total (95% CI)**			**2467**			**2109**	**27.0%**	**−3.13 [−7.51; 1.26]**
**Prediction interval**			.			.	**--**	**[−18.90; 12.65]**
Heterogeneity: $Tau^2 = 27.3045$; $Chi^2 = 59.59$, df = 5 ($P < 0.01$); $I^2 = 92\%$								
**Total (95% CI)**			**10081**			**8665**	**100.0%**	**−2.42 [−4.43; −0.41]**
**Prediction interval**								**[−12.21; 7.37]**
Heterogeneity: $Tau^2 = 20.8326$; $Chi^2 = 332.92$, df = 20 ($P < 0.01$); $I^2 = 94\%$								
Test for subgroup differences: $Chi^2 = 1.25$, df = 2 ($P = 0.54$)								

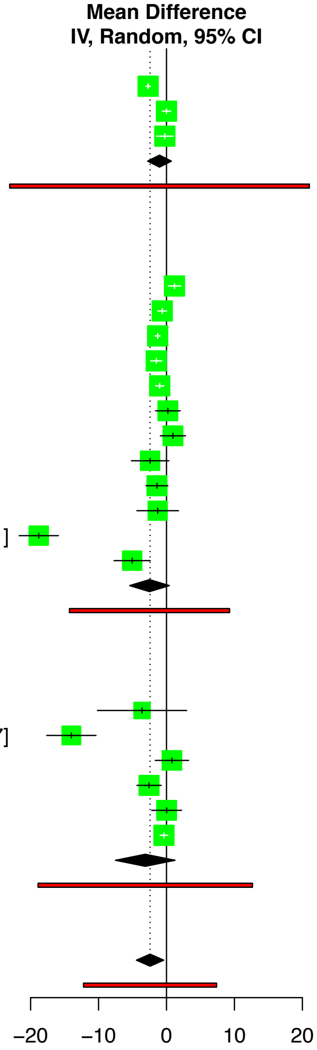

Supplementary Figure 13. Subgroup analysis for hospital length of stay stratified by alerting purpose.

The size of the green square indicates the weight of each individual study. The black diamond represents the pooled effect size for each subgroup as well as for the overall effect. The red bars represent the prediction interval.

Abbreviations: SD = standard deviation; CI = confidence interval; IV = inverse variance.

Study or Subgroup	Experimental Events	Experimental Total	Control Events	Control Total	Weight	Risk Ratio IV, Random, 95% CI
**Design = SIRS**						
Semler 2015	30	218	30	189	5.3%	0.87 [0.54; 1.38]
Hooper 2012	31	220	23	222	5.1%	1.36 [0.82; 2.26]
Gatewood 2015	14	122	18	137	4.3%	0.87 [0.45; 1.68]
Narayanan 2016	18	103	20	111	4.7%	0.97 [0.54; 1.73]
Umscheid 2015	41	545	52	595	5.7%	0.86 [0.58; 1.27]
Arabi 2017a	60	195	208	436	6.5%	0.64 [0.51; 0.81]
Arabi 2017b	118	699	208	436	6.6%	0.35 [0.29; 0.43]
Austrian 2018	91	1306	71	838	6.2%	0.82 [0.61; 1.11]
Benson 2014	20	116	19	123	4.7%	1.12 [0.63; 1.98]
Berger 2010	46	890	52	908	5.7%	0.90 [0.61; 1.33]
Hayden 2016	18	130	10	108	3.9%	1.50 [0.72; 3.10]
Manaktala 2017	19	464	105	1170	5.2%	0.46 [0.28; 0.73]
McRee 2014	1	96	7	75	1.0%	0.11 [0.01; 0.89]
Threatt 2020	9	145	72	165	4.3%	0.14 [0.07; 0.27]
Lipatov 2022	203	1096	166	854	6.6%	0.95 [0.79; 1.15]
**Total (95% CI)**		**6345**		**6367**	**75.7%**	**0.72 [0.53; 0.96]**
Heterogeneity: $Tau^2 = 0.2646$; $Chi^2 = 107.74$, df = 14 ($P < 0.01$); $I^2 = 87\%$						
**Design = MEWS**						
Guirgis 2017	202	1568	226	1637	6.7%	0.93 [0.78; 1.11]
Croft 2014	16	119	15	65	4.4%	0.58 [0.31; 1.10]
Na 2021	231	957	218	639	6.7%	0.71 [0.61; 0.83]
**Total (95% CI)**		**2644**		**2341**	**17.8%**	**0.78 [0.61; 0.99]**
Heterogeneity: $Tau^2 = 0.0272$; $Chi^2 = 6.22$, df = 2 ($P = 0.04$); $I^2 = 68\%$						
**Design = qSOFA**						
Song 2019	93	315	118	316	6.5%	0.79 [0.63; 0.99]
**Total (95% CI)**		**9304**		**9024**	**100.0%**	**0.73 [0.59; 0.91]**
**Prediction interval**						**[0.29; 1.83]**
Heterogeneity: $Tau^2 = 0.1775$; $Chi^2 = 119.01$, df = 18 ($P < 0.01$); $I^2 = 85\%$						
Test for subgroup differences: $Chi^2 = 0.31$, df = 2 ($P = 0.86$)						

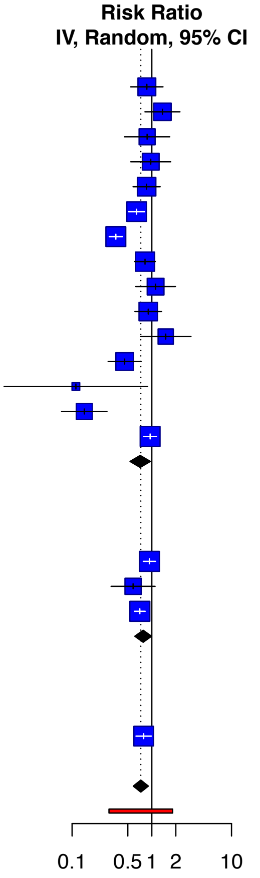

Supplementary Figure 14. Subgroup analysis for mortality stratified by different rules to alert sepsis onset.

The size of the green square indicates the weight of each individual study. The black diamond represents the pooled effect size for each subgroup as well as for the overall effect. The red bars represent the prediction interval.

Abbreviations: SD = standard deviation; CI = confidence interval; IV = inverse variance; SIRS = systematic inflammatory response syndrome; qSOFA = quick Sepsis Related Organ Failure Assessment; MEWS = Modified Early Warning Score.
